# Polyamine signaling communications play a key role in regulating the pathogenicity of *Dickeya fangzhongdai*

**DOI:** 10.1128/spectrum.01965-23

**Published:** 2023-10-24

**Authors:** Congcong Xie, Weihan Gu, Zhongqiao Chen, Zhibin Liang, Shufen Huang, Lian-Hui Zhang, Shaohua Chen

**Affiliations:** 1 National Key Laboratory of Green Pesticide, Guangdong Province Key Laboratory of Microbial Signals and Disease Control, South China Agricultural University Integrative Microbiology Research Centre, Guangzhou, China; 2 Guangdong Laboratory for Lingnan Modern Agriculture, Guangzhou, China; Institute of Microbiology, Chinese Academy of Sciences, Beijing, China

**Keywords:** *Dickeya fangzhongdai*, polyamines, bacterial motility, plant cell wall-degrading enzymes, pathogenicity

## Abstract

*Dickeya fangzhongdai* is a devastating bacterial pathogen infecting a wide range of crops and ornamental plants worldwide. As a newly identified bacterial species in 2016, the regulatory mechanisms that govern its virulence are still a mystery. In this study, we explored the potential roles of polyamine-mediated cell-to-cell communication in regulation of *D. fangzhongdai* virulence. Null mutation of *speA* and *speC* in *D. fangzhongdai* strain ZXC1, which encodes polyamine biosynthesis through arginine and ornithine pathways, respectively, dramatically reduced bacterial motility, decreased production of plant cell wall degradation (PCWD) enzymes, and attenuated the bacterial virulence on taro and potato. We then tested the effect of various polyamine molecules in the restoration of the mutant phenotypes and showed that putrescine was the most potent signal in the regulation of virulence traits in strain ZXC1. In addition, we found that taro extract contained active signals to rescue putrescine-deficient phenotypes. High-performance liquid chromatography mass spectrometry analysis validated the *speA* was essential for production of putrescine in *D. fangzhongdai* ZXC1. We further showed that the putrescine transporters PotF and PlaP are required for putrescine-mediated cell-to-cell communication and virulence against taro and potato tubers. quantitative reverse transcription-PCR analysis demonstrated that putrescine influences the pathogenicity of *D. fangzhongdai* ZXC1 by regulating the expression of PCWD enzymes, bacterial chemotaxis, and flagellar-related genes. The findings from this study shed a new light for elucidating the pathogenic mechanisms of *D. fangzhongdai* and present useful clues for developing relevant disease control strategies.

**IMPORTANCE:**

*Dickeya fangzhongdai* is a newly identified plant bacterial pathogen with a wide host range. A clear understanding of the cell-to-cell communication systems that modulate the bacterial virulence is of key importance for elucidating its pathogenic mechanisms and for disease control. In this study, we present evidence that putrescine molecules from the pathogen and host plants play an essential role in regulating the bacterial virulence. The significance of this study is in (i) demonstrating that putrescine signaling system regulates *D. fangzhongdai* virulence mainly through modulating the bacterial motility and production of PCWD enzymes, (ii) outlining the signaling and regulatory mechanisms with which putrescine signaling system modulates the above virulence traits, and (iii) validating that *D. fangzhongdai* could use both arginine and ornithine pathways to synthesize putrescine signals. To our knowledge, this is the first report to show that putrescine signaling system plays a key role in modulating the pathogenicity of *D. fangzhongdai*.

## INTRODUCTION

The genus *Dickeya* is among the 10 most important bacterial phytopathogens in the world (1). Since the establishment of the genus *Dickeya* gen. nov. in 2005 with 6 species (2), the genus has now been expanded to 12 species, including *Dickeya paradisiaca*, *Dickeya dianthicola*, *Dickeya dadantti*, *Dickeya zeae*, *Dickeya chrysanthemi*, *Dickeya aquatica*, *Dickeya solani*, *Dickeya fangzhongdai*, *Dickeya undicila*, *Dickeya lacustri*s, *Dickeya poaceiphila*, and *Dickeya oryzae* (2 – 10). Among them, *D. fangzhongdai* was isolated from diseased pear tree (*Pyrus pyrifolia*) and identified as a new species for the first time in 2016 (5), and is the only *Dickeya* pathogen that can infect woody plant. To date, about 27 *D. fangzhongdai* strains have been isolated and identified, infecting both monocotyledonous and dicotyledonous plants, including pear trees, taro [*Colocasia esculenta* (L.) Schott], *Allium cepa L*, *Allium fistulosum* L., *Phalaenopsis orchid*, *Aglaonema* sp., *Artocarpus heterophyllus* Lam., *Dendrobium nobile*, Banxia (*Pinellia ternata*), and banana (*Musa* spp.) (11 – 22). These findings suggest that *D. fangzhongdai* might have formidable virulence and corresponding regulatory mechanisms to outperform and infect a wide range of host organisms.

Most studies on the pathogenic mechanisms of *Dickeya* pathogens were focused on *Dickeya dadantii* and *Dickeya oryzae. D. dadantii* infects and cause various plant soft rot diseases by producing an array of virulence factors, including cell wall-degrading enzymes, bacterial motility, extracellular polysaccharide, blue pigment indigoidine, iron assimilation system, and type III secretion system (23). The above virulence traits are also well conserved in *D. oryzae*, which, in addition, also produces a family of phytotoxins known as zeamines (24, 25). Noticeably, zeamines and bacterial motility were identified as the two key virulence determinants of *D. oryzae*. The zemine-minus mutants of *D. oryzae* could hardly cause infection in rice (25, 26), whereas its motility mutants were significantly retarded in invasion and systemic infection of the same plant (27 – 29). Virulence factor production was controlled by several cell-to-cell communication systems, including the acyl-homoserine lactone-mediated quorum sensing (QS) system (26, 30 – 32), Vfm QS system (33, 34), and putrescene signaling system (27). Significantly, *D. oryzae* could detect and respond to both host and itself produced putrescene as an intraspecies and interkingdom cell-to-cell communication signal to activate expression of various virulence genes (27). In contrast, as a newly identified bacterial pathogen, hardly any work has been done in characterization of the virulence and regulatory mechanisms in *D. fangzhongdai*.

Taro is an edible tropical crop belonging to the Araceae family, which is widely distributed throughout the world and has high nutritional, ornamental, medicinal, and economic values (35). However, its plantation has been severely affected by bacterial soft rot disease caused by various bacterial pathogens (36). We recently found that *D. fangzhongdai* is one of the major pathogens causing taro soft rot disease in Guangdong Province of China (13). Bioinformatics analysis showed that polyamine synthesis and transporter genes are highly conserved in *D. fangzhongdai*, suggesting that putrescine might also play a role in regulating the virulence and pathogenic mechanisms of this newly discovered *Dickeya* species. In this study, to explore the role of putrescine in the regulation of *D. fangzhongdai* virulence, we performed a systematic deletion analysis of the genes involved in polyamine biosynthesis and transportation by using the taro isolate ZXC1 as the parental strain. The results unveiled the key roles of putrescine biosynthesis in supporting bacterial growth, production of plant cell wall-degrading enzymes, and bacterial motility. In addition, we also demonstrated that *D. fangzhongdai* ZXC1 was able to influx exogenous putrescine through its specific transporters, PotF and PlaP, to compensate for the defected phenotypes due to deletion of the genes responsible for putrescine biosynthesis. The findings from this study add a new insight on the pathogenic mechanisms of *D. fangzhongdai*, which may facilitate developing new strategies to curb the rampage of this important bacterial pathogen.

## RESULTS

### Bioinformatic analysis of the genes involved in polyamine synthesis and transportation

We firstly conducted bioinformatic analysis of the putative genes associated with polyamine biosynthesis in *D. fangzhongdai* ZXC1. Five polyamine biosynthesis genes have been identified and characterized in the well-studied *Escherichia coli*, including *speA*, encoding an arginine decarboxylase [National Center for Biotechnology Information (NCBI) accession number NP_417413.1]; *speB*, encoding an agmatinase (NCBI accession number NP_417412.1); *speC*, encoding an ornithine decarboxylase (NCBI accession number NP_417440.4); *speD*, encoding a S-adenosylmethionine decarboxylase (NCBI accession number NP_414662.1); and *speE*, encoding a spermidine synthase (NCBI accession number NP_414663.1) (37). In addition, in *Yersinia pestis*, *aguA*, encoding an agmatine deiminase (NCBI accession number AJJ88030.1), and *aguB*, encoding an N-carbamoylputrescine amidase (NCBI accession number AJJ88068.1), are also known to be involved in polyamine biosynthesis (37). Among them, SpeA and SpeB are involved in conversion of arginine to produce putrescine (38); SpeC uses ornithine as a substrate to generate putrescine (38). In *Y. pestis* and *Pseudomonas aeruginosa*, where SpeB is missing, arginine is firstly converted to agmatine by SpeA, and then agmatine deiminase AguA catalyzes formation of N-carbamoylputrescine, which is further converted into putrescine by amidinohydrolase AguB. SpeE catalyzes formation of spermidine by addition of the aminopropyl moiety derived from decarboxylated S-adenosylmethionine (dSAM), which is synthesized by SpeD (39, 40). A Blast search using the above seven genes, respectively, against the genome sequence of *D. fangzhongdai* ZXC1 (NCBI number CP119773.1) led to identification of all the genes encoding polyamine biosynthesis homologs except *speB*. These homologs at amino acid level are most similar to their counterparts in *D. dadantii* 3937, followed by *D. oryzae* EC1, two *Yersinia pestis* strains, *P. aeruginosa* PAO1, and *E. coli* K-12 MG 1655 (Table 1). It seems that *D. fangzhongdai* ZXC1 contains all the genes required for polyamine biosynthesis, and its biosynthetic pathway is reminiscent of that of *Y. pestis* (37) and *P. aeruginosa* PAO1 (41).

**TABLE 1:** Analysis of the genes encoding polyamine biosynthesis and transportation in *D. fangzhongdai* ZXC1^*a*^

Strains (sequence accession number in NCBI)	Identity (similarity) in amino acid level to the corresponding homologs fund in ZXC1 (%)							
	speA	AguA	AguB	speC	speD	speE	potF	plaP
*Dickeya dadantii* 3937 (CP002038.1)	99.85	96.71	98.30	99.02	98.48	97.56	98.64	–
*Dickeya oryzae* EC1 (CP006929.1)	97.26	93.41	97.96	95.40	98.86	95.82	98.92	97.78
*Escherichia coli* str. K-12 MG 1655 (NC_000913.3)	86.32	–	–	73.29	84.09	78.60	79.46	81.33
*Yersinia pestis* CO92 (CP009973.1)	87.86	77.71	92.52	–	90.15	83.51	84.01	–
*Yersinia pestis* KIM10+ (AE009952.1)	87.86	–	–	74.58	90.15	83.51	84.01	–
*Pseudomonas aeruginosa* PAO1 (NC_002516.2)	44.52	55.62	64.60	31.06	69.50	61.37	58.47 (SpuD)	–

^*a*^ “–” indicates the gene was not found in the corresponding genome homologs.

To date, four putrescine-specific transporters (PotE, PotFGHI, PlaP, and PuuP) and one spermidine/putrescine transporter (PotABCD) have been identified and characterized in *E. coli*, in which PotE, PotF, PlaP, PuuP, and PotD are corresponding substrate-binding proteins (42 – 45). Among them, PotF and PlaP are involved in transportation of putrescine signal molecules in *D. oryzae* EC1 (27). While bioinformatics analysis did not reveal PotE and PuuP homologs, highly conserved homologs of PotF and PlaP from *D. fangzhongdai* ZXC1 were identified, which displayed high levels of similarities to the corresponding counterparts from *D. dadantii* 3937, *D. oryzae* EC1, *Y. pestis* strains, *P. aeruginosa* PAO1, and *E. coli* K-12 MG 1655 (Table 1). In addition, three copies of the *potD* gene encoding potential spermidine/putrescine transporter are present in the genome of *D. fangzhongdai* ZXC1, whose products are similar to the PotD proteins from *D. dadantii* 3937 and *D. oryzae* EC1, respectively, but displayed low levels of similarities to the corresponding proteins from *Y. pestis* strains, *P. aeruginosa* PAO1, and *E. coli* K-12 MG 1655 (Table S4).

### Effect of null mutation of polyamine synthesis genes on extracellular enzyme production and bacterial motility

To study the role of polyamines in *D. fangzhongdai* ZXC1 physiology and pathogenicity, polyamine synthase genes *speA*, *aguA*, *aguB*, *speC*, *speD*, and *speE* were deleted in-frame and named as △*A*, △*uA*, △*uB*, △*C*, △*D*, and△*E*, respectively. Given that both *speA* and *speC* are involved in the synthesis of putrescine, their double-deletion mutant △*AC* was also generated for phenotype analysis. The growth assay showed that the growth rate of the single mutant △*A*, △*uA*, △*uB*, △*C*, △*D*, and △*E* in Luria-Bertani (LB) medium or minimal medium (MM) cultures was consistent with that of wild-type *D. fangzhongdai* ZXC1 (Fig. S1a through d). In addition, the growth rate of double-mutant △*AC* in the LB medium was consistent with that of wild-type *D. fangzhongdai* ZXC1, while the growth rate of the double-mutant △*AC* in the MM was delayed compared to that of wild-type strain ZXC1 (Fig. S1c and d).

**Fig 1:**
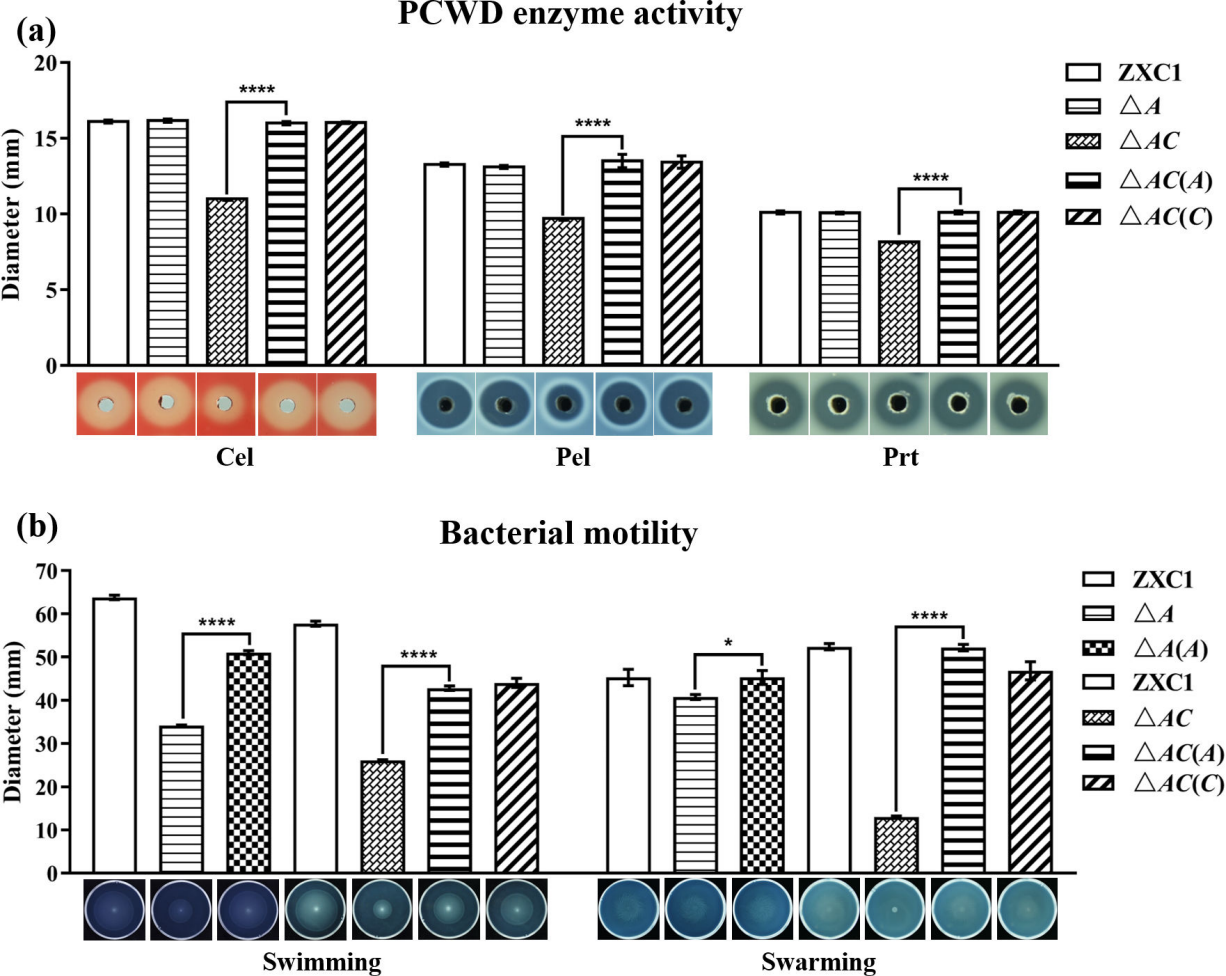
Analysis of PCWD enzyme activity and bacterial motility in wild-type *D. fangzhongdai* ZXC1 and derivatives. (**a**) PCWD enzyme activity assay. (**b**) Bacterial swimming and swarming motility assay. Symbols: △*A*, *speA* in-frame deletion mutant; △*AC*, *speA* and *speC* double-eletion mutant; △*A*(*A*), △*A* complemented with *speA*; △*AC*(*A*) and △*AC*(*C*), △*AC* complemented with *speA* or *speC*, respectively. The data shown are the mean ± standard error (*n* = 3 independent experiments). Statistical significance: *P＜0.05, ****P＜0.0001. ns, not significant (by Student’s *t*-test).

Similarly, the single mutants △*A*, △*uA*, △*uB*, △*C*, △*D*, and △*E* cultured in corresponding substrate media (Table S2) produced basically the same levels of plant cell wall degradation (PCWD) enzymes, including cellulase (Cel), pectinase (Pel), and protease (Prt), as their wild-type strain ZXC1 (Fig. 1a; Fig. S2a). In contrast, the double-mutant △*AC* showed significantly decreased activity of three PCWD enzymes, compared with wild-type *D. fangzhongdai* ZXC1. In *trans* expression of either *speA* or *speC* in the double-mutant △*AC* could fully rescue the mutant phenotypes (Fig. 1a).

We then analyzed the cell motility of *D. fangzhongdai* ZXC1 and its derivatives. The single-deletion mutants △*C*, △*D*, and △*E* did not show obvious difference in both swimming and swarming motility compared with strain ZXC1, and mutants △*uA* and △*uB* showed a moderately weakened swimming motility (Fig. S2b). Intriguingly, however, the mutant △*A* showed significantly decreased swimming motility and slightly decreased swarming motility (Fig. 1b) when compared with wild-type *D. fangzhongdai* ZXC1 and its complement △*A*(*A*). Deletion of *speC* in the background of △*A* further decreased the bacterial swimming and swarming motility (Fig. 1b). Taken together, the above data suggest the pathogen could use both SpeA and SpeC pathways to synthesize putrescine to modulate bacterial growth, PCWD enzyme production, and bacterial motility.

### Impact of exogenous addition of polyamines and taro tissue extract on bacterial motility

**Fig 2:**
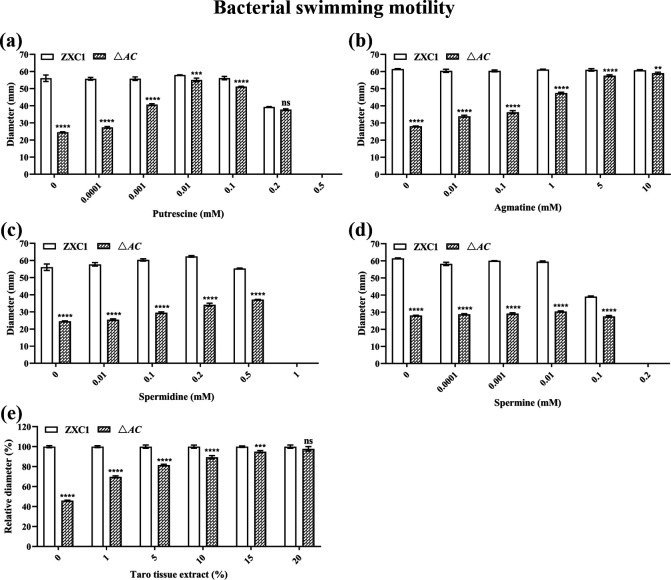
Effect of exogenous polyamines and taro tissue extract on the swimming motility of *D. fangzhongdai* ZXC1 and double-mutant △*AC*, respectively. Bacterial motility with or without putrescine (**a**), agmatine (**b**), spermidine (**c**), and spermine (**d**) were determined 16 h post inoculation. The bacterial swimming motility with or without taro extract was recorded 10 h post inoculation and expressed as relative values by setting the values of strain ZXC1 as 100 for the convenience of comparison (**e**). the data shown are the mean ± standard error (*n* = 3). Statistical significance: **P＜0.01, ***P＜0.001, ****P＜0.0001. ns, not significant (by two-way analysis of variance with multiple comparisons).

To verify the regulatory role of polyamines in *D. fangzhongdai* ZXC1, we tested the effect of polyamines on restoration of swimming motility in double-mutant △*AC*. As shown in Fig. 2, putrescine showed the best effect, which could fully restore the swimming motility of double-mutant △*AC* to the wild-type level at a final concentration of 0.01 mM (Fig. 2a). However, further increasing putrescine level appeared detrimental, and the swimming motility of strain ZXC1 and double-mutant △*AC* was completely inhibited at a final concentration of 0.5-mM putrescine (Fig. 2a). Agmatine was at least over 100 times less effective than putrescine in restoration of the mutant phenotype, but no side effect was noted on both wild-type ZXC1 and the double-mutant △*AC* even at a concentration as high as 10 mM (Fig. 2b). Spermidine and spermine had no effect or merely a minor effect in activation of swimming motility and could block bacterial motility at 1.0 and 0.2 mM, respectively (Fig. 2c and d). The above results indicate that putrescine is the key cell-to-cell communication signal produced by *D. fangzhongdai* ZXC1 in regulation of its physiology and virulence.

We then tested whether taro tissue may contain sufficient signal to restore the defected phenotype of the double-mutant △*AC*. Since taro extract contains rich nutrients, strain ZXC1 and mutant in the swimming plate supplemented with taro extract grew faster than on the control plate without taro extract, and hence the bacterial motility was recorded 10 h instead of normally 16 h post inoculation (Fig. S3). For the convenience of comparison, the mutant motility was expressed as a relative value to that of wild type, which was arbitrarily set as 100 (Fig. 2e). With the exogenous addition of 20% taro tissue extract, the swimming motility of double-mutant △*AC* was recovered to about 98% of that of *D. fangzhongdai* ZXC1 (Fig. 2e). In conclusion, the above data suggest that putrescine is the key signal regulating bacterial swimming motility in *D. fangzhongdai* ZXC1, and that the pathogen appeared able to tape the host signal to activate its pathogenic mechanisms.

### Disruption of *speA* and *speC* abolishes putrescine production in strain ZXC1

**Fig 3:**
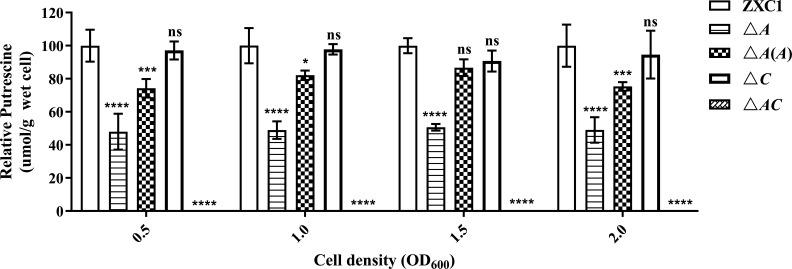
Quantitative measurement of cellular putrescine content in wild-type *D. fangzhongdai* ZXC1, the single-deletion mutants △*A* and △*C*, the double-deletion mutant △*AC*, and complemented strain *A*(*A*) at different growth stages. △*C* is the *speC* in-frame deletion mutant. The data shown are the mean ± standard error (*n* = 3). Statistical significance: *P＜0.05, ***P＜0.001, ****P＜0.0001. ns, not significant (by two-way analysis of variance with multiple comparisons).

The above data suggest that putrescine is the cell-to-cell communication signal in modulation of bacterial physiology and production of PCWD enzymes. We therefore determine the cellular concentration of polyamines in *D. fangzhongdai* ZXC1 and its derivatives. The cell-free extracts of bacterial strains were prepared for derivation of polyamine molecules using benzoyl chloride (46, 47). The concentration of benzoylated polyamines were determined using high-performance liquid chromatography coupled with mass spectrometry (HPLC-MS) with reference to standard benzoylated polyamines. The results showed that deletion of *speA* led to about 40%–50% decrease in putrescine level compared to wild-type *D. fangzhongdai* ZXC1 at different bacterial growth stages, which could be partially rescued by in *trans* expression of wild-type *speA* (Fig. 3). Intriguingly, null mutation of *speC* did not seem to significantly alter the putrescine level, whereas double deletion of both *speA* and *speC* almost fully abolished putrescine production (Fig. 3). Quantitative reverse transcription-PCR (qRT-PCR) analysis showed that expression of *speA* was increased in the mutant △*C* except at low cell density with OD_600_ at 0.5 (Fig. S4a). In contrast, *speC* expression was increased from OD_600_ at 0.5–1.5, but then decreased with OD_600_ at 2.0 in the mutant △*A* (Fig. S4b).

Considering that putrescine is also a host signal for host-pathogen cell-to-cell communication (27), we then determine its level along with that of other polyamines in taro tuber tissues. The results showed that the concentrations of putrescine and spermidine in the taro tissue extracts were about 16.3 and 8.7 nmol/g, respectively, while spermine could not be detected. The findings indicate that putrescine is likely to be the major polyamine compound in taro tubers.

### Effect of null mutation of polyamine transporter genes on PCWD enzyme production and bacterial motility

Bacteria not only can synthesize polyamines by themselves but also can also absorb exogenous polyamines through specific polyamine transporters. The bioinformatics analysis showed that *D. fangzhongdai* ZXC1 genome contains the genes encoding putrescine transporters PotF and PlaP, respectively (Table 1). To verify the roles of PotF and PlaP in putrescine-mediated cell-to-cell communication among *D. fangzhongdai* ZXC1 cells and between the bacterial cells and host organisms, single- and double-deletion mutants of *potF* and *plaP* were generated in the genetic background of wild-type *D. fangzhongdai* ZXC1 and the *speA* mutant △*A* and named as △*F*, △*P*, △*FP*, △*AF*, △*AP*, and △*AFP*, respectively. In addition, a quadruple-deletion mutant △*ACFP* was also generated by deleting *speC* in the genetic background of the triple-deletion mutant △*AFP*. The growth curve showed that the growth rates of double-mutant △*AC* and triple-mutant △*AFP* in LB or MM were similar to that of wild-type *D. fangzhongdai* ZXC1, while the growth rate of quadruple mutant △*ACFP* in LB and MM was substantially retarded compared to that of strain ZXC1 (Fig. S1c and S1d). Intriguingly, when 0.1-mM putrescine was added into the LB or MM, the growth rate of mutant △*ACFP* was partially rescued (Fig. S1e and S1f), suggesting that there could be an unidentified putrescine transporter in *D. fangzhongdai* ZXC1.

**Fig 4:**
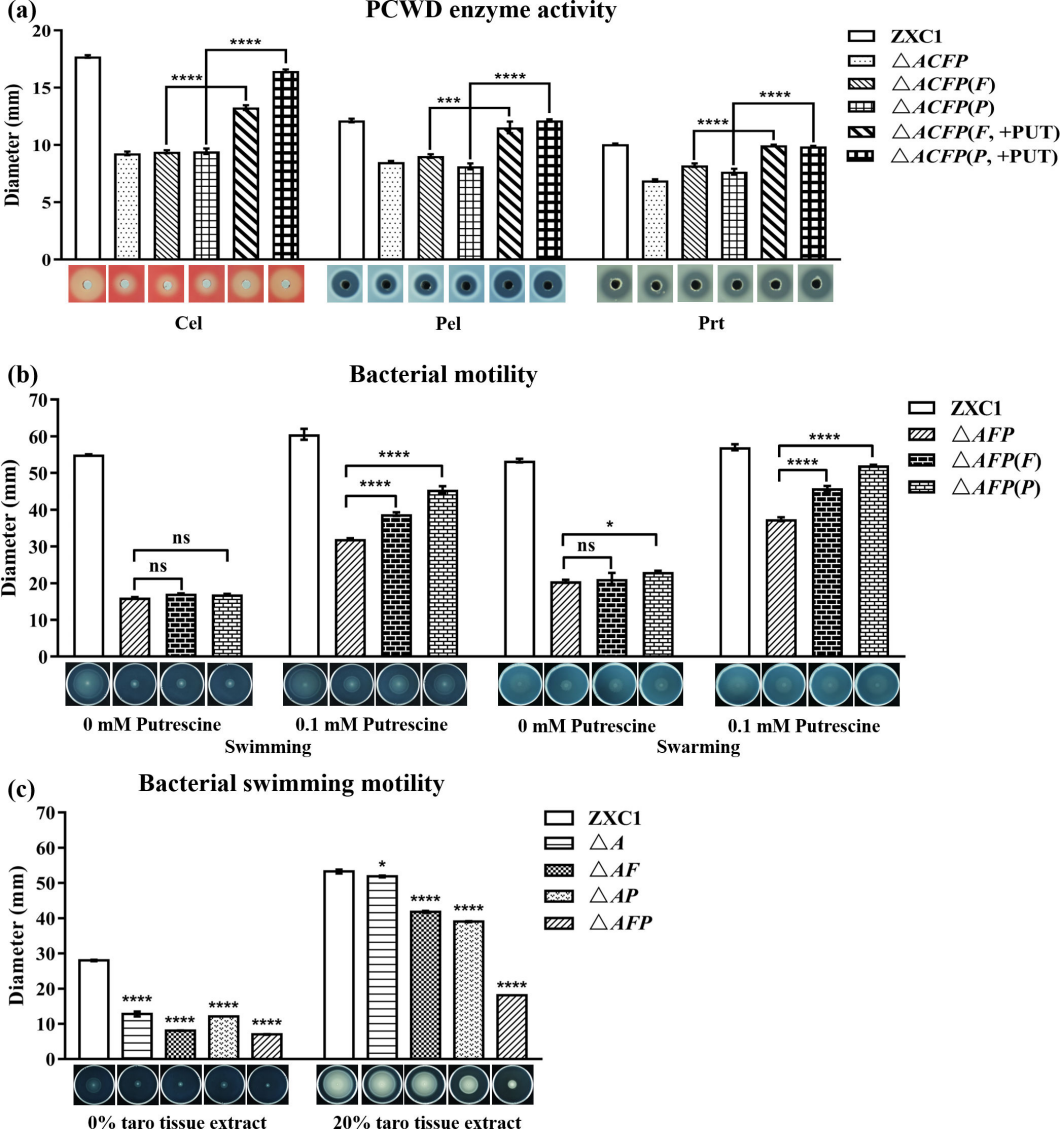
Analysis of PCWD enzyme production and bacterial motility in wild-type *D. fangzhongdai* ZXC1, △*ACFP*, △*AFP*, and its complemented strains △*ACFP*(*F*), △*ACFP*(*P*), *AFP*(*F*), and △*AFP*(*P*) with or without addition of 0.1-mM putrescine. (**a**) PCWD enzyme assay of strain ZXC1 and derivatives with or without putrescine. ***P＜0.001, ****P＜0.0001 (by Student’s *t*-test); (**b**) Bacterial motility assay of strain ZXC1 and derivatives with or without putrescine. *P＜0.05, ****P＜0.0001. ns, not significant (by two-way analysis of variance with multiple comparisons). (**c**) Bacterial motility assay of strain ZXC1 and derivatives with or without taro extract. Symbols: △*AFP*, the triple-deletion mutant of *speA*, *potF*, and *plaP*; △*ACFP*, the quadruple-deletion mutant of *speA*, *speC*, *potF*, and *plaP*; △*AFP*(*F*) and △*AFP*(*P*), △*AFP* complemented with *potF* and *plaP,* respectively; △*ACFP*(*F*) and △*ACFP*(*P*), △*ACFP* complemented with *potF* and *plaP*, respectively. The data shown are the mean ± standard error (*n* = 3). Statistical significance: *P＜0.05, ****P＜0.0001 (by Student’s *t*-test).

While production of PCWD enzymes in the △*F*, △*P*, △*FP*, △*AF*, △*AP*, and △*AFP* mutants in the MM was similar to that of wild-type ZXC1 and their parental mutant △*A* (Fig. S5a and b), PCWD enzyme activities in quadruple mutant △*ACFP* were significantly decreased compared with wild-type ZXC1 and its complemented strains △*ACFP*(*F*) and △*ACFP*(*P*) supplemented with 0.1-mM putrescine (Fig. 4a). In the motility assay, we found that the bacterial swimming motility of △*F*, △*P*, and △*FP* mutants were similar to that of wild-type ZXC1 (Fig. S5c). In contrast, the bacterial motility of quadruple mutant △*ACFP* was significantly reduced compared to the wild-type *D. fangzhongdai* ZXC1 and mutants △*A* and △*AC* (Fig. S6). While the bacterial swimming motility of mutant △*AFP* was significantly decreased compared with that of wild-type *D. fangzhongdai* ZXC1, exogenous addition of 0.1 mM putrescine to its complemented △*AFP*(*F*) and △*AFP*(*P*) could partially restore the defected motility (Fig. 4b). Similarly, we found that deletion of transporter genes *potF* and *plaP* in the genetic background of *speA* mutant △*A* abolished its response to taro extract in restoration of defected motility (Fig. 4c). In conclusion, the above data suggest that PotF and PlaP are essential for *D. fangzhongdai* ZXC1 to uptake exogenous putrescine.

### Quantitative RT-PCR analysis of the virulence genes regulated by putrescine

To understand how putrescine could affect PCWD enzyme production and cell motility of *D. fangzhongdai* ZXC1, qRT-PCR analysis was performed to determine the expression levels of the genes associated with PCWD enzymes and bacterial motility. The genes encoding PCWD enzyme production and bacterial motility and associated regulators were identified by Blast searches using the corresponding homologs from the well-characterized *D. dadantii* 3937 (48). This led to identification of 43 genes in *D. fangzhongdai* ZXC1, which share >83% identity at amino acid level with corresponding homologs in *D. dadantii* 3937 (Table S5).

**Fig 5:**
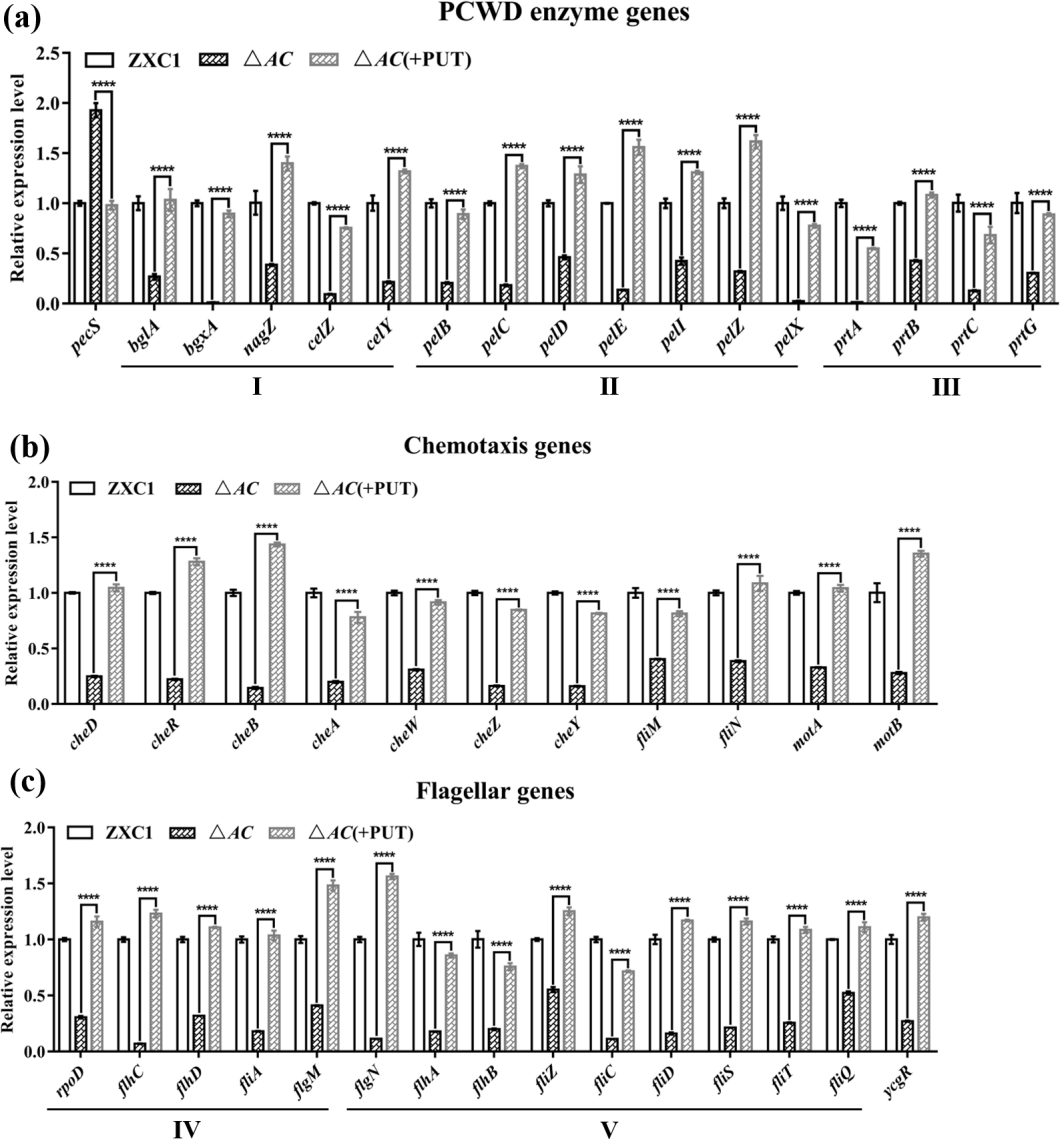
qRT-PCR analysis of the genes involved in the production of PCWD enzymes and bacterial motility in *D. fangzhongdai* ZXC1, double-mutant △*AC*, and △*AC* with 0.1-mM putrescine. (**a**) qRT-PCR analysis of the PCWD genes: I, genes encoding cellulases; II, genes encoding pectate lyases; and III, genes encoding proteases. (**b**) qRT-PCR analysis of the genes related to bacterial chemotaxis: *cheD*, *cheR*, *cheB*, *cheA*, *cheW*, *cheZ*, and *cheY* encode bacterial chemotaxis proteins; *fliM* and *fliN* encode flagellar motor switch proteins; and *motA* and *motB* encode flagellar motor proteins. (**c**) qRT-PCR analysis of the genes related to bacterial flagellar biogenesis. IV, flagellar regulator genes; *rpoD* encodes the RNA polymerase global regulator factor (σ70), which works in cooperation with FlhDC to bind to the promoter regions of flagellar and non-flagellar operons to activate the expression of class II flagellar genes (51); *flhC* and *flhD* encode class I master flagellar transcriptional activators (52); *fliA* encodes the class II RNA polymerase sigma factor (σ28) to transcribe class III flagellar genes (53); *flgM* encodes the *fliA*-specific negative regulator factor of flagellin synthesis (54). V, the genes encoding flagellar biosynthetic proteins; *ycgR* encodes a flagellar Brake protein, which regulates swimming and swarming in a c-di-GMP-dependent manner (55). The macerated areas of potato and taro were measured at 24 and 48 h post inoculation, respectively, and the data shown are the mean ± standard error (*n* = 3). Statistical significance: ****P＜0.0001 (by two-way analysis of variance with multiple comparisons).

Wild-type *D. fangzhongdai* ZXC1 and mutant △*AC* were cultured in MM, and RNA samples were prepared for qRT-PCR analysis when bacterial cell density (OD_600_) reached about 1.0. As shown in Fig. 5a; Table S5, expression levels of the PCWD enzyme genes, including *bglA*, *bgxA*, *nagZ*, *celY*, and *celZ*, related to cellulase biosynthesis (49); *pelB*, *pelC*, *pelD*, *pelE*, *pelI*, *pelZ,* and *pelX*, associated with pectinase biosynthesis (49); and *prtA*, *prtB*, *prtC*, and *prtG*, those encoding proteases (49), in mutant △*AC* were significantly reduced compared with wild-type ZXC1 and the mutant △*AC* supplemented with 0.1-mM putrescine. Similarly, expression levels of chemotaxis genes (https://www.kegg.jp/pathway/map02030), including *cheD*, *cheR*, *cheB*, *cheA*, *cheZ*, *cheY*, and *cheW*, associated with bacterial chemotaxis (50), *fliM*, *fliN*, *motB*, and *motA*, encoding flagellar motor proteins (28), in mutant △*AC* were markedly reduced compared with wild-type ZXC1 and the mutant △*AC* supplemented with 0.1-mM putrescine (Fig. 5b; Table S5). And the expression levels of the flagellar genes (https://www.kegg.jp/pathway/map02040), including *flgN*, *flhA*, *flhB*, *fliZ*, *fliC*, *fliD*, *fliS*, *fliT*, and *fliQ*, encoding flagellar biosynthesis proteins (28), and the *ycgR* gene, encoding flagellar brake protein (28), in mutant △*AC* were markedly reduced compared with wild-type ZXC1 and the mutant △*AC* supplemented with 0.1-mM putrescine (Fig. 5c; Table S5).

We then compared the transcriptional expression levels of the genes encoding regulatory functions. As shown in Fig. 5a, *pecS* encodes a negative transcriptional regulator of PCWD enzyme production (56, 57), and its transcript level in mutant △*AC* was two times higher than that in wild-type ZXC1 or the mutant △*AC* supplemented with 0.1-mM putrescine. Interestingly, although the growth rate of △*pecS* was significantly lower than that of wild-type *D. fangzhongdai* ZXC1 in MM, production of PCWD enzymes was much higher than wild-type *D. fangzhongdai* ZXC1 (Fig. S7a and S7b), suggesting that the negative regulator plays a key role in curbing the expression of PCWD enzyme genes under the *in vitro* conditions used in this study. It is worth noting that this study found for the first time that PecS also negatively regulated the expression of the genes encoding proteases in *D. fangzhongdai* ZXC1. The *cheB* encodes a chemotaxis response regulator protein-glutamate methylesterase and is known to play a role in regulation of methylation of bacterial chemotaxis genes (58). The growth rate of △*cheB* was consistent with that of wild-type *D. fangzhongdai* ZXC1 in MM, while its motility was decreased by 50% (Fig. S7a and S7c). Expression levels of the genes encoding transcriptional regulators of the flagellar operon (*rpoD*, *flhC*, *flhD*, *fliA*, and *flgM*) in mutant △*AC* were decreased by 70%–90% compared to those in mutant △*AC* supplemented with 0.1-mM putrescine (Fig. 5c), suggesting their roles in positive regulation of flagellar biogenesis. Taken together, the above findings suggest that putrescine modulates the expression of the genes involved in PCWD enzyme production by regulating the expression of negative transcriptional regulator gene *pecS* and regulates bacterial motility by affecting the expression of genes related to bacterial chemotaxis, flagellar biosynthesis, and flagellar motors in *D. fangzhongdai* ZXC1.

### The mutants defective in putrescine-mediated cell-to-cell communication were much attenuated in virulence against taro and potato

**Fig 6:**
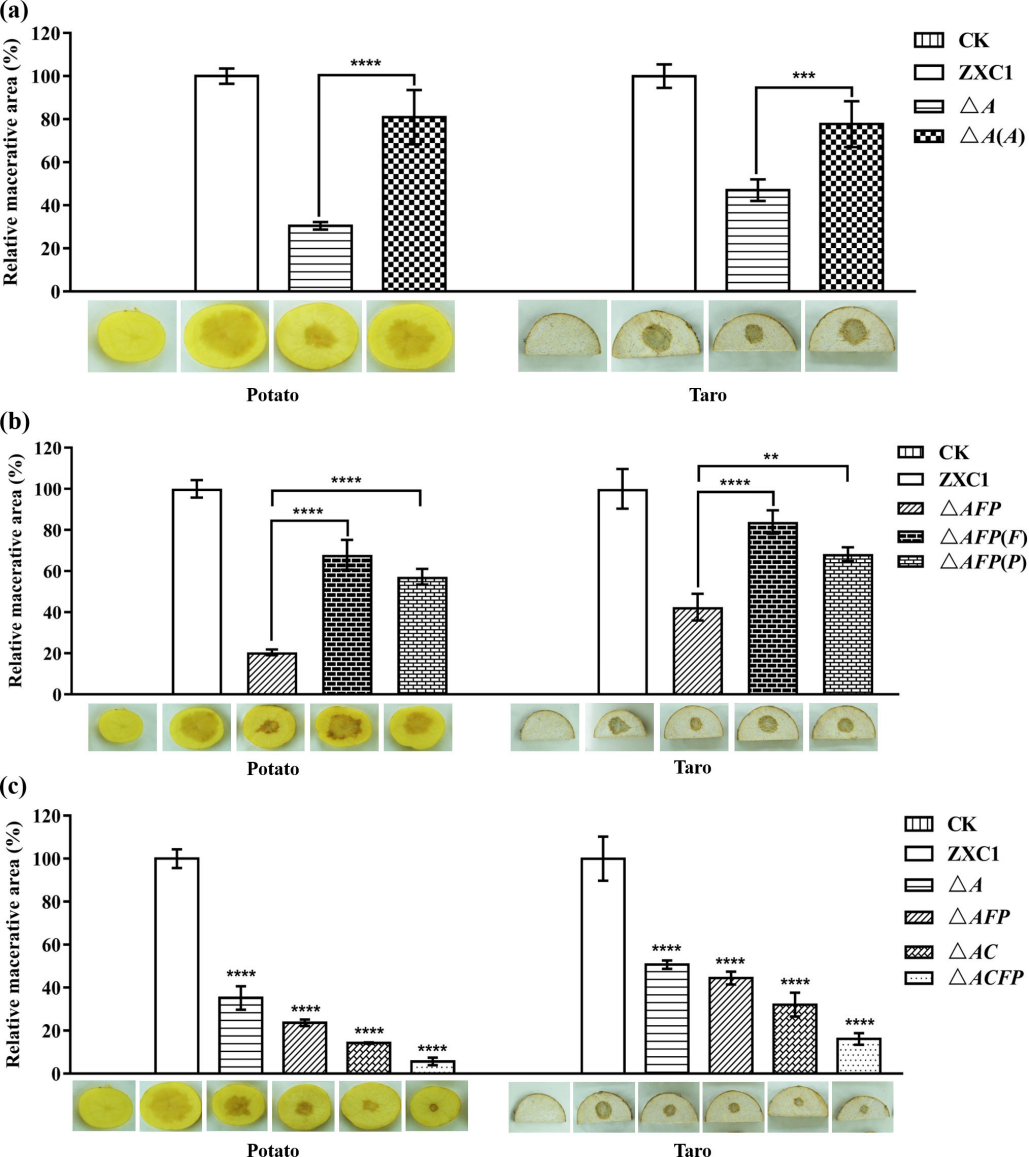
Virulence of *D. fangzhongdai* ZXC1 and derivatives on taro and potato. (**a**) Virulence assay with mutant △*A* and strain ZXC1 on potato and taro tubers; (**b**) virulence assay with mutant △*AFP* and strain ZXC1 on potato and taro tubers; (**c**) virulence assay with single-, double-, triple-, and quadruple-deletion mutant strain ZXC1 on potato and taro tubers. The data shown are the mean ± standard error (*n* = 3). Statistical significance: **P＜0.01, ***P＜0.001, ****P＜0.0001 (by two-way analysis of variance with multiple comparisons).

Bacterial virulence assay was performed on taro and potato using wild-type *D. fangzhongdai* ZXC1, putrescine-related mutants, and complemented strains. The bacterial cells were cultured in MM overnight with OD_600_ adjusted to about 0.5, and 2 µL of which was added to the center of taro or potato tubers. The results showed that *D. fangzhongdai* ZXC1 could infect both dicotyledonous (potato) and monocotyledonous (taro) plants (Fig. 6). Compared with the wild-type strain, the macerated zones of △*A* in potato and taro were reduced by about 70% and 53%, respectively, which could be partially rescued by in *trans* expression of the wild-type *speA* in the mutant (Fig. 6a). To validate the role of putrescine transporters *in planta*, we then compared the virulence of △*AFP* and strain ZXC1. The results showed that the macerated zones of △*AFP* were reduced by about 80% and 58%, respectively, compared to wild-type ZXC1, and complementation with *potF* or *plaP* could restore the mutant virulence (Fig. 6b). The bacterial virulence of quadruple mutant △*ACFP* was further reduced compared with the single-deletion mutant △*A*, double-deletion mutant △*AC*, and triple-deletion mutant △*AFP* (Fig. 6c). These results demonstrate the critical role of putrescine-mediated cell-to-cell communication in coordination of *D. fangzhongdai* virulence.

## DISCUSSION

*D. fangzhongdai* is a newly emerged and defined plant pathogen which was found capable of infecting numerous host plants (5, 13 – 15, 18 – 22), but its pathogenic and corresponding regulatory mechanisms are mostly unknown. In this study, we used the *D. fangzhongdai* strain ZXC1 isolated from taro, as a model organism to investigate the role of putrescine in *D. fangzhongdai* physiology and virulence. The findings from this study demonstrated that the pathogen could tape to the putrescine molecules produced by itself and those from host organisms as a signal to regulate production of PCWD enzymes and bacterial motility. The *D. fangzhongdai* mutants defective in putrescine signaling communication were drastically attenuated in its virulence against taro and potato. Our data also outlined the molecular mechanisms by which the putrescine signaling system modulates the bacterial motility and PCWD enzyme production.

In contrast to *D. oryzae* EC1, which produces putrescine mainly through the arginine pathway (27), we found that *D. fangzhongdai* ZXC1 could use both arginine and ornithine pathways to synthesize putrescine signals. Bioinformatics analysis indicated that *D. fangzhongdai* ZXC1 encodes highly conserved arginine decarboxylase SpeA and ornithine decarboxylase SpeC, which share over 95% identity with *D. oryzae* at amino acid level (Table 1). HPLC-MS analysis results showed that the intracellular putrescine level of the *speA* deletion mutant △*A* was reduced by 50% when compared with *D. fangzhongdai* ZXC1 (Fig. 3), whereas double deletion of both *speA* and *speC* in *D. fangzhongdai* ZXC1 abolished putrescine biosynthesis (Fig. 3). Interestingly, transcriptional expression of *speA* was upregulated in the *speC* deletion mutant, and vice versa, deletion of *speA* also led to increased expression of *speC* (Fig. S4). Highly agreeable with the key role of putrescine in keeping with bacterial growth (Fig. S1) and virulence (Fig. 6), these findings suggest that *D. fangzhongdai* might have a compensation mechanism to balance the expression of *speA* and *speC* so as to maintain the cellular level of putrescine, which is worthy of further investigations.

Spermidine, spermine, and putrescine constitute a group of ubiquitous aliphatic small polycationic molecules known as polyamines, which are widely distributed from bacteria to plants and animals. Bacterial pathogens could tape on to these polyamines through polyamine transporters or sensors (59, 60). Among these polyamine molecules, putrescine was the most effective signal in rescuing the *speA* mutant phenotypes (Fig. 2). In this regard, it is interesting to note that putrescine was the most abundant polyamine molecule in taro extract. Further analysis found that two highly conserved putrescine transporters in *D. fangzhongdai* ZXC1, i.e., PotF and PlaP (Table 1), play a crucial role in influx of exogenous putrescine signals into the bacterial cells, including those from taro tissues (Fig. 4). Similarly, PlaP and PotF were also found in transporting putrescine into *D. oryzae* cells (27), suggesting that these putrescine transportation systems could be well conserved in the *Dickeya* genus. However, unlike *D. oryzae* EC1 that relies solely on PlaP and PotF in efflux of putrescine signals (27), our data suggest that *D. fangzhongdai* ZXC1 might contain additional polyamine transporters, which could uptake both spermidine and putrescine in the absence of PlaP and PotF (Fig. 4c). Our preliminary work seemed to preclude the PotABCD transporter, which is known for uptaking both spermidine and putrescine (61), as the swimming motility of the *speA*/*plaP*/*potF*/*potD* deletion mutant was still rescuable by exogenous addition of 0.1-mM putrescine (Fig. S8). This intriguing potential transporter needs to be further identified and characterized.

**Fig 7:**
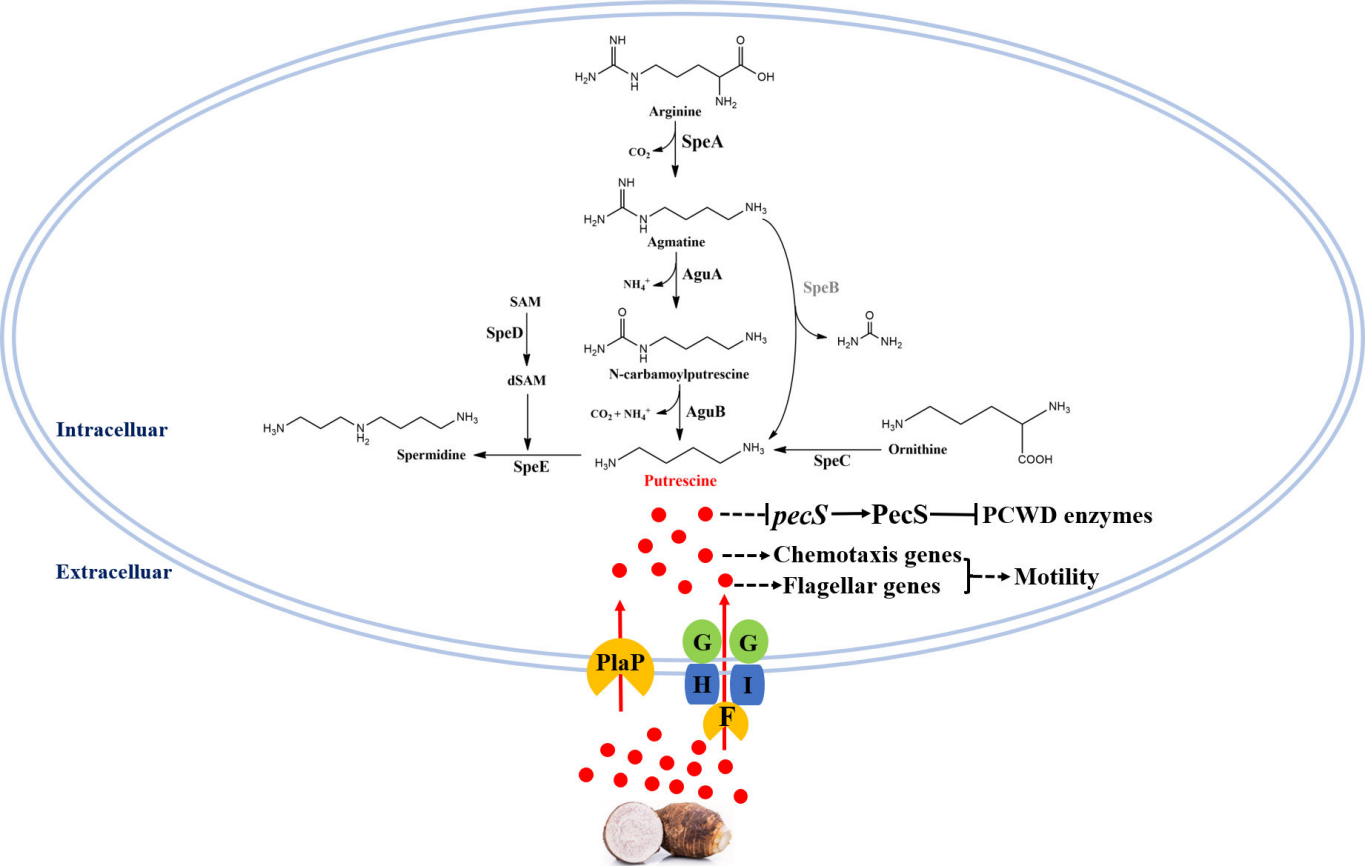
Putrescine signal regulates the PCWD enzyme production and bacterial motility of *D. fangzhongdai*.

Putrescine signaling system was firstly found regulating *D. oryzae* virulence and systemic infection through modulating motility and biofilm formation (27). Similarly, deletion of *speA* and *speC* in *D. fangzhongdai* ZXC1 drastically attenuated its swimming and swarming motility (Fig. 1). However, we were not able to determine whether putrescine system could regulate biofilm formation in *D. fangzhongdai* ZXC1 as the pathogen did not form biofilms under the same experimental conditions used for *D. oryzae* analysis (27). Interestingly, in contrast to *D. oryzae* in which putrescine signal was not involved in the regulation of PCWD enzyme production (27), deletion of *speC* and *speA* significantly reduced the production of PCWD enzymes, and the defected phenotypes could be rescued via expression of the corresponding wild type genes in mutants or by exogenous addition of putrescine or taro tissue extract (Fig. 1 and 2). In addition, deletion of *speA* in *D. oryzae* did not alter the bacterial growth rate (27), whereas putrescine was needed for supporting *D. fangzhongdai* growth (Fig. S1). Our findings from this study thus add new functions to the list of putrescine regulatory spectrum in plant bacterial pathogens (Fig. 7).

The evidence of polyamines as signaling molecules in microorganisms has been accumulating in recent years, including spermidine from mammalian host that induces the expression of the genes encoding type III secretion system in a human bacterial pathogen *Pseudomonas aeruginosa* (60, 62); putrescine that regulates bacterial motility in *Proteus mirabilis* (45, 63), *E. coli* K-12 (44), *D. oryzae* EC1 (64), and *D. zeae* MS3 (65); and biofilm formation and disassembly in *Yersiniapestis* and *Shewanella oneidensis*, respectively (66, 67). However, the molecular mechanisms by which polyamine signals modulate various phenotypes remain largely unknown. Bearing this in mind, we specifically analyzed the genes encoding and regulating PCWD enzyme production and bacterial motility using qRT-PCR. The results suggest that putrescine might modulate the production of PCWD enzymes through downregulating the expression of PecS (Fig. 5a), which is a negative regulator known for curbing the expression of PCWD enzyme genes (56), and that putrescine could promote *D. fangzhongdai* motility by upregulating the genes encoding bacterial chemotaxis and flagellar biogenesis (28) (Fig. 5b and c), because bacterial motility is mainly guided and driven by chemotaxis and flagellar (68, 69).

In summary, the findings from this study unveiled that *D. fangzhongdai* has evolved a four-component putrescine signaling system containing two signal synthases and two signal transporters to regulate bacterial virulence by inducing the expression of the genes encoding PCWD enzymes and bacterial motility (Fig. 7). In brief, the putrescine signal molecules produced by the SpeA and SpeC pathways in *D. fangzhongdai* and those from host plants activate PCWD enzyme production by downregulation of the negative regulator gene *pecS* and induce the transcriptional expression of chemotaxis and flagellar synthesis genes to modulate bacterial motility, and hence influence the bacterial virulence. In this signaling process, the bacterial transporter PotF and PlaP play a key role in uptaking extracellular putrescine signal into the bacterial cells for modulation of target gene expression (Fig. 7). This is highly aggregable with the critical roles of putrescine signaling system for the bacterial survival and competition, as such dual configurations would offer the pathogen sufficient flexibility and plasticity to accommodate complicated and adverse circumstances. We further present evidence of how the putrescine signaling system could modulate PCWD enzyme production and bacterial motility. These original findings add a new insight on the biological functions of polyamine signaling systems and present a solid basis and useful clues for further elucidating the putrescine signaling network and mechanisms of action in the regulation of *D. fangzhongdai* physiology and virulence.

## MATERIALS AND METHODS

### Bacterial strains, growth conditions, and reagents

The bacterial strains and plasmids used and constructed in this study are listed in Table S1. The medium formula used in this study is shown in Table S2. *Escherichia coli* strains were maintained in LB medium at 37°C, and *D. fangzhongdai* ZXC1 and its derivatives were grown at 28°C in LB or MM and used for comparison of bacterial growth rate. Putrescine was purchased from Macklin; agmatine sulfate salt, spermidine, and spermine were purchased from Sigma-Aldrich. Antibiotics were added at the following final concentrations when required: kanamycin, 50 µg/mL; streptomycin, 50 µg /mL; and tetracycline, 10 µg/mL.

### Generation of in-frame deletion mutants and complemented strains

In-frame deletion mutants of *speA*, *aguA*, *aguB*, *speC*, *speD*, and *speE* were generated through homologous recombination (25, 26) and named as △*A*, △*uA*, △*uB*, △*C*, △*D*, and△*E*, respectively. In addition, double-deletion mutants △*AC*, △*AF*, and △*AP* were generated by deleting *speC*, *potF*, and *plaP* in the genetic background of △*A*, respectively. A triple-deletion mutant △*AFP* was generated by deleting *plaP* in the genetic background of the double-deletion mutant △*AF*. A quadruple-deletion mutant △*ACFP* mutant was generated by deleting *speC* in the genetic background of the triple-deletion mutant △*AFP*. Detailed descriptions of the strains and mutants are provided in Table S1. All the primers used in this study are listed in Table S3. Firstly, fragments containing about 500 bp upstream (primers: gene-1 and gene-2) and downstream regions (primers: gene-3 and gene-4) of the corresponding target gene were amplified using *D. fangzhongdai* ZXC1 chromosomal DNA as template, respectively. Secondly, the upstream and downstream fragments of the target gene were fused together by using primer pair gene-1 and gene-4. The fusion fragment was then ligated to the suicide plasmid pKNG101 digested with restriction enzymes *BamH* I, and transformed into competent cells of *E. coli* CC118λ. The recombinant constructs were verified by DNA sequencing, and introduced into *D. fangzhongdai* ZXC1 by tri-parental mating to generate in-frame mutants as previously described (25).

To generate complemented strains, the encoding regions of *speA*, *speC*, *potF*, and *plaP* were amplified from *D. fangzhongdai* ZXC1 genomic DNA using the primers C-gene-F and C-gene-R by the primers listed in Table S3, respectively. The PCR products were ligated to expression vector PLAFR3 digested with restriction enzyme *BamH* I and transformed into competent cells of *E. coli* DH5α. The recombinant constructs were verified by DNA sequencing and introduced into corresponding mutants by tri-parental mating and confirmed by PCR analysis.

### Bacterial extracellular enzyme activity assay

Cel, Pel, and Prt enzymes were assayed using carboxymethyl cellulose sodium, polygalacturonic acid, and non-fat-dried milk as substrates (Table S2), respectively, as described previously (70). Briefly, assay plates were prepared by pouring about 40 mL of substrate medium into a 13 × 13 cm square Petri dishes and allowing it to set at room temperature until completely solidified. The wells with 5 mm in diameter were punched in the assay plates with a metal puncher. The bacteria were inoculated in MM and cultured at 28°C and 200 rpm until population density reached about OD_600_ = 1.0. Bacterial cells were removed by centrifugation with 12,000 rpm at 4°C for 5 min. Then, 20 µL of bacterial supernatants was taken and added into the wells of the assay plates, which were air-dried and invertedly incubated at 28°C. After incubation for about 17 h (Cel) and 32 h (Pel and Prt), respectively, the plates were stained with dye as follows: Cel assay plates were stained with 0.1% Congo red for 25 min and then decolored with NaCl (1 M) for 30 min twice; Pel assay plates were treated with HCl (1 M) for coloration; and the transparent zones surrounding the wells in Prt assay plates were directly recorded. The experiments were repeated three times in triplicates.

### Bacterial motility assay

Bacterial motility assay was carried out as follows: 1.5-µL fresh cultures of *D. fangzhongdai* ZXC1 and its derivatives were spotted, respectively, on the center of a 90-mm semisolid plate containing 15-mL swimming medium (Table S2) and were incubated at 28°C. Swarming motility was assayed under the same conditions, except that bacterial cells were spotted onto a 90-mm semisolid swarming plate containing 15-mL swarming medium (Table S2) and incubated at 28°C. The diameters of swimming or swarming motility were measured 16 h post inoculation. To test the effect of polyamines and taro extract on restoration of the defected swimming motility of the polyamine biosynthesis and transportation mutants, taro extracts were prepared by processing the peeled taro with a juicer, filtered by eight layers of gauze, centrifuged by 12,000 rpm at 4°C for 1 h, and then filtered by a 0.45-µm filter to obtain taro extracts. The swimming motilities of the *D. fangzhongdai* ZXC1 and its mutants were quantified using the method described above by adding putrescine, spermidine, spermine, and taro extract to the swimming medium at a final concentration of 0.1, 0.5, and 0.1 mM and 20%, respectively. The above experiments were repeated three times in triplicate.

### Polyamine derivatization and quantification

The cellular levels of polyamines in *D. fangzhongdai* ZXC1 and its derivatives were measured based on previous described methods with minor modifications (27, 46, 47, 71, 72). Briefly, bacterial cells were grown in MM to OD_600_ = 0.5, 1.0, 1.5, and 2.0, respectively, at 28°C with shaking at 200 rpm. Aliquots of 10 mL of the bacterial cell suspension were centrifuged for 15 min at 4°C and 4,000 rpm; the bacterial pellets were collected and the wet weight of bacterial cells was recorded. The cell pellets were washed once with 10-mL ddH_2_O, resuspended in 1-mL lysis buffer (xTractor Buffer and xTractor Buffer Kit, purchased from Takara), and then incubated for 3 h at 4°C. The supernatants were collected by centrifugation and used for derivatization. To 400 µL of bacterial supernatants, 2 mL of 2-M NaOH solution and 14-µL of benzoyl chloride were added, shaken for 2 min, and incubated for 30 min at 37°C (27, 47). The benzoylated mixtures were added to 4 mL of saturated NaCl solution and shaken for 20 s, which were then extracted by adding 4-mL petroleum ether. The petroleum ether phase was dried at room temperature. The samples were dissolved by adding 500-µL methanol filtrated with a 0.22-µm filter. The benzoylated polyamines were assayed with HPLC-MS using a 1.8-µm Eclipse Plus C18 (Agilent) column fitted with a 100 × 2.1-mm guard column at a flow rate of 0.2 mL/min. Mass spectroscopy (Agilent 6540B Q-TOF) was used to verify the identity of each peak observed in the HPLC fractions. A standard curve was generated using various concentrations of benzoylated putrescines. The polyamine levels in the taro extract were measured using the same methods. The experiments were repeated three times in triplicate.

### qRT-PCR assay

Bacterial strains were cultured in fresh MM (with or without 0.1-mM putrescine) at 28°C to OD_600_ = 1.0. RNA samples were prepared using an SV total RNA isolation system kit (Promega). RNA quantity was measured using a NanoDrop (Wilmington, DE, USA) ND-100 spectrophotometer, and RNA integrity was determined by using agarose gel electrophoresis. Total RNA samples were treated with DNase I to remove DNA contaminations and then reverse transcribed into double-strand cDNA using HiScript III RT SuperMix for qPCR (Vazyme). Quantitative PCR (qPCR) was performed using ChamQ Universal SYBR qPCR Master Mix (Vazyme) with the qPCR primers listed in Table S3. The experiments were repeated three times in triplicate.

### Bacterial pathogenicity assay

Pathogenicity assays of *D. fangzhongdai* and derivatives were performed using potato and taro tubers. Briefly, potato and taro tubers were washed and air dried, and slices about 5 mm in thickness were prepared. A small hole was made using a sterilized needle in the center of potato and taro tuber slices, to which 2 µL of bacterial suspension with OD_600_ at 0.5 about 4.35 × 10^7^ CFU/mL was added using a sterilized pipette tip. The inoculated plant tuber slices were incubated at 28°C and 80% relative humidity, and soft rot symptoms were recorded at 24 or 48 h post inoculation as indicated. The experiments were repeated three times in triplicate. The sizes of maceration zones in plant slices were measured using ImageJ.

## SUPPLEMENTAL MATERIAL

Tables S1-S5, Fig. S1-S8 spectrum.01965-23-s0001.docx Table S1-S5 (Bacterial strains and plasmids used in this study, the medium formula used in this study, primers used in this study, similarity of *D. fangzhongdai* ZXC1 putative polyamine transporter substrate-binding protein PotD with its homologs in other bacterial species, and analysis of the genes encoding PCWD enzymes, motility and corresponding regulators in *D. fangzhongdai* ZXC1), and Fig. S1-S8 (growth curves of wild-type *D. fangzhongdai* ZXC1 and its derivatives in LB and MM medium [with or without 0.1 mM putrescine]; analysis of PCWD enzyme activity and bacterial motility in wild-type *D. fangzhongdai* ZXC1 and polyamine synthesis gene mutants; effect of taro tissue extract on the swimming motility of *D. fangzhongdai* ZXC1 and mutant △A; qRT-PCR analysis of speA or speC transcript level in the background of mutant △C [a] or △A [b], respectively; analysis of PCWD enzyme activity and bacterial motility in wild-type *D. fangzhongdai* ZXC1 and mutant △A, △AF, △AP, △AFP, △F, △P and △FP; analysis bacterial motility in wild-type *D. fangzhongdai* ZXC1 and mutant △A and quadruple mutant △ACFP; impact of pecS and cheB mutation on *D. fangzhongdai* ZXC1 growth, PCWD enzyme production and motility; analysis of bacterial swimming motility in wild-type *D. fangzhongdai* ZXC1 and putative polyamine transporter substrate-binding protein PotD mutants with or without 0.1 mM putrescine).

ASM does not own the copyrights to Supplemental Material that may be linked to, or accessed through, an article. The authors have granted ASM a non-exclusive, world-wide license to publish the Supplemental Material files. Please contact the corresponding author directly for reuse.
