# Supplementary material for: Polyamine signaling communications play a key role in regulating the pathogenicity of Dickeya fangzhongdai
Source: Microbiol Spectr. 2023 Oct 24;11(6):e01965-23. doi: 10.1128/spectrum.01965-23 (PMC10715095; doi:10.1128/spectrum.01965-23)
Supplement: Tables S1-S5, Fig. S1-S8 — Table S1-S5 (Bacterial strains and plasmids used in this study, the medium formula used in this study, primers used in this study, similarity of D. fangzhongdai ZXC1 putative polyamine transporter substrate-binding protein PotD with its homologs in other bacterial species, and analysis of the genes encoding PCWD enzymes, motility and corresponding regulators in D. fangzhongdai ZXC1), and Fig. S1-S8 (growth curves of wild-type D. fangzhongdai ZXC1 and its derivatives in LB and MM medium [with or without 0.1 mM putrescine]; analysis of PCWD enzyme activity and bacterial motility in wild-type D. fangzhongdai ZXC1 and polyamine synthesis gene mutants; effect of taro tissue extract on the swimming motility of D. fangzhongdai ZXC1 and mutant △A; qRT-PCR analysis of speA or speC transcript level in the background of mutant △C [a] or △A [b], respectively; analysis of PCWD enzyme activity and bacterial motility in wild-type D. fangzhongdai ZXC1 and mutant △A, △AF, △AP, △AFP, △F, △P and △FP; analysis bacterial motility in wild-type D. fangzhongdai ZXC1 and mutant △A and quadruple mutant △ACFP; impact of pecS and cheB mutation on D. fangzhongdai ZXC1 growth, PCWD enzyme production and motility; analysis of bacterial swimming motility in wild-type D. fangzhongdai ZXC1 and putative polyamine transporter substrate-binding protein PotD mutants with or without 0.1 mM putrescine). [file spectrum.01965-23-s0001.docx]

**Supporting Material**

**Polyamine signaling communications play a key role in regulating the pathogenicity of *Dickeya fangzhongdai***

Congcong Xie^1^, Weihan Gu^1^, Zhongqiao Chen^1^, Zhibing Liang^1^, Shufen Huang^1^, Lian-Hui Zhang^1,2*^ and Shaohua Chen^1*^

^1^National Key Laboratory of Green Pesticide, Guangdong Province Key Laboratory of Microbial Signals and Disease Control, Integrative Microbiology Research Centre, South China Agricultural University, Guangzhou 510642, China

^2^Guangdong Laboratory for Lingnan Modern Agriculture, Guangzhou 510642, China

* Corresponding authors at: Integrative Microbiology Research Centre, South China Agricultural University, Guangzhou 510642, China.

E-mail address: [lhzhang01@scau.edu.cn](mailto:lhzhang01@scau.edu.cn) and [shchen@scau.edu.cn](mailto:shchen@scau.edu.cn).

**Supplementary Tables:**

**Table S1**. Bacterial strains and plasmids used in this study.

**Table S2.** The medium formula used in this study.

**Table S3.** Primers used in this study.

**Table S4.** Similarity of *D. fangzhongdai* ZXC1 putative polyamine transporter substrate-binding protein PotD with its homologs in other bacterial species.

**Table S5.** Analysis of the genes encoding PCWD enzymes, motility and corresponding regulators in *D. fangzhongdai* ZXC1.

**Supplementary Figures:**

**Fig. S1** Growth curves of wild-type *D. fangzhongdai* ZXC1 and its derivatives in LB and MM medium (with or without 0.1 mM putrescine).

**Fig. S2** Analysis of PCWD enzyme activity and bacterial motility in wild-type *D. fangzhongdai* ZXC1 and polyamine synthesis genes mutants.

**Fig. S3** Effect of taro tissue extract on the swimming motility of *D. fangzhongdai* ZXC1 and mutant △*A*.

**Fig. S4** qRT-PCR analysis of *speA* or *speC* transcript level in the background of mutant △*C* (a) or △*A* (b), respectively.

**Fig. S5** Analysis of PCWD enzyme activity and bacterial motility in wild-type *D. fangzhongdai* ZXC1 and mutant △*A*, △*AF*, △*AP*, △*AFP*, △*F*, △*P* and △*FP*.

**Fig. S6** Bacterial motility assay of wild-type *D. fangzhongdai* ZXC1. mutant △*A* and quadruple mutant △*ACFP*.

**Fig. S7** Impact of *pecS* and *cheB* mutation on *D. fangzhongdai* ZXC1 growth, PCWD enzyme production and motility.

**Fig. S8** Bacterial swimming motility assay of wild-type *D. fangzhongdai* ZXC1 and putative polyamine transporter PotD mutants with or without 0.1 mM putrescine.

**Table S1.** Strains and plasmids used in this study.

| **Strains or plasmid** | **Relevant phenotypes and characteristics ^a^** | **Source or reference** |
| --- | --- | --- |
| ***Dickeya fangzhongdai*** |  |  |
| ZXC1 | Wild type of *Dickeya fangzhongdai* ZXC1 | Lab collection |
| ∆*A* | *speA* deletion mutant derived from ZXC1 | This study |
| ∆*uA* | *aguA* deletion mutant derived from ZXC1 | This study |
| ∆*uB* | *aguB* deletion mutant derived from ZXC1 | This study |
| ∆*C* | *speC* deletion mutant derived from ZXC1 | This study |
| ∆*D* | *speD* deletion mutant derived from ZXC1 | This study |
| ∆*E* | *speE* deletion mutant derived from ZXC1 | This study |
| ∆*AC* | *speC* deletion mutant derived from ∆*A* | This study |
| ∆*F* | *potF* deletion mutant derived from ZXC1 | This study |
| ∆*P* | *plaP* deletion mutant derived from ZXC1 | This study |
| ∆*FP* | *potF* deletion mutant derived from ∆*P* | This study |
| ∆*AF* | *potF* deletion mutant derived from ∆*A* | This study |
| ∆*AP* | *plaP* deletion mutant derived from ∆*A* | This study |
| ∆*AFP* | *potF* deletion mutant derived from ∆*AP* | This study |
| ∆*ACFP* | *speC* deletion mutant derived from ∆*AFP* | This study |
| ∆*A*(*A*) | The complemented strain of ∆*A*, Tc ^r^ | This study |
| ∆*AC*(*A*) | The complemented strain of ∆*AC*, Tc ^r^ | This study |
| ∆*AC*(*C*) | The complemented strain of ∆*AC*, Tc ^r^ | This study |
| ∆*AFP*(*F*) | The complemented strain of ∆*AFP*, Tc ^r^ | This study |
| ∆*AFP*(*P*) | The complemented strain of ∆*AFP*, Tc ^r^ | This study |
| ∆*ACFP*(*F*) | The complemented strain of ∆*ACFP*, Tc ^r^ | This study |
| ∆*ACFP*(*P*) | The complemented strain of ∆*ACFP*, Tc ^r^ | This study |
| ∆*pecS* | *pecS* deletion mutant derived from ZXC1 | This study |
| ∆*cheB* | *cheB* deletion mutant derived from ZXC1 | This study |
| ***Escherichia coli*** |  |  |
| CC118 | Host for plasmid constructs derived from pKNG101 | Lab collection |
| DH5α | deoR, recA, endA, hsdR, supE, thi, gyrA, relA, | TransGen Biotech |
| pRK2013 | *Thr leu thi recA hsdR hsdM pro*, Km ^r^ | Lab collection |
| **Plasmids** |  |  |
| pKNG101 | Knockout vector, Str^r^ | Lab collection |
| pKNG101-*speA* | pKNG101 containing in-frame  deleted fragement of *speA*, Str ^r^ | This study |
| pKNG101-*aguA* | pKNG101 containing in-frame  deleted fragement of *aguA*, Str ^r^ | This study |
| pKNG101-*aguB* | pKNG101 containing in-frame  deleted fragement of *aguB*, Str ^r^ | This study |
| pKNG101-*speC* | pKNG101 containing in-frame  deleted fragement of *speC*, Str ^r^ | This study |
| pKNG101-*speD* | pKNG101 containing in-frame  deleted fragement of *speD*, Str ^r^ | This study |
| pKNG101-*speE* | pKNG101 containing in-frame  deleted fragement of *speE*, Str ^r^ | This study |
| pKNG101-*potF* | pKNG101 containing in-frame  deleted fragement of *potF*, Str ^r^ | This study |
| pKNG101-*plaP* | pKNG101 containing in-frame  deleted fragement of *plaP*, Str ^r^ | This study |
| pKNG101-*pecS* | pKNG101 containing in-frame  deleted fragement of *pecS*, Str ^r^ | This study |
| pKNG101-*cheB* | pKNG101 containing in-frame  deleted fragement of *cheB*, Str ^r^ | This study |
| PLAFR3 | expression vector, Tc ^r^ | Lab collection |
| PLAFR3- *speA* | PLAFR3 containing *speA*  encoding region at the its promoter, Tc ^r^ | This study |
| PLAFR3- *speC* | PLAFR3 containing *speC*  encoding region at the its promoter, Tc ^r^ | This study |
| PLAFR3- *potF* | PLAFR3 containing *potF*  encoding region at the its promoter, Tc ^r^ | This study |
| PLAFR3- *plaP* | PLAFR3 containing *plaP*  encoding region at the its promoter, Tc ^r^ | This study |

Km ^r^, Str ^r^ or Tc ^r^ = resistance to kanamycin, streptomycin or tetracycline respectively.

**Table S2.** The medium formula used in this study.

| **Medium name** | **Component** | **Content** |
| --- | --- | --- |
| **LB medium** | Tryptone  Yeast extract  NaCl | 10.0 g/L  5.0 g/L  10.0 g/L |
| **MM medium** | K_2_HPO_4_  KH_2_PO_4_  (NH_4_)_2_SO_4_  Mannitol  Glycerin  MgSO_4_·7H_2_O  CaCl_2_  FeSO_4_  MnCl_2_ | 10.5 g/L  4.5 g/L  2.0 g/L  2.0 g/L  2.0 g/L  0.2 g/L  0.01 g/L  0.005 g/L  0.002 g/L |
| **Enzyme detection medium** |  |  |
| **Cellulase** | Sodium carboxymethylcellulose  Na_3_PO_4_  Agarose | 1.0 g/L  3.8 g/L  8.0 g/L |
| **Pectate lysae** | Polygalacturonic acid  Tris  CaCl_2_  Agarose | 5.0 g/L  4.8448 g/L  0.1125 g/L  8.0 g/L |
| **Protease** | Tryptone  NaCl  Nonfat-Dried milk  Agar powder | 10.0 g/L  10.0 g/L  1%  15.0 g/L |
| **Bacterial motility detection medium** |  |  |
| **Swimming** | Bacto-peptone  NaCl  Bacto-agar | 5.0 g/L  5.0 g/L  3.0 g/L |
| **Swarming** | Tryptone  NaCl  Agarose | 10.0 g/L  5.0 g/L  5.0 g/L |

**Table S3.** Primers used in this study.

| Primers | Description | Sequence |
| --- | --- | --- |
| **Used generating deletion mutants** |  |  |
| *speA*-1 | Forward primer for upstream of *speA* | 5’-GTCGAC***ggatcc***CCGGGTACCATACAGACGGAAAAGACAGCCAGCG-3’ |
| *speA*-2 | Reverse primer for upstream of *speA* | 5’-GATCGCCATGTCTGACGACGAATAATATTTCGTACGG-3’ |
| *speA*-3 | Forward primer for downstream of *speA* | 5’-CCGTACGAAATATTATTCGTCGTCAGACATGGCGATC-3’ |
| *speA*-4 | Reverse primer for downstream of *speA* | 5’-CTTATGGTACCCGGGGATCCTTGTCCGTAATTCGGGCGATTCCG-3’ |
| *aguA*-1 | Forward primer for upstream of *aguA* | 5’-GTCGAC***ggatcc***CCGGGTACCGGCAACCTCGATAAACCGGTAGTG-3’ |
| *aguA*-2 | Reverse primer for upstream of *aguA* | 5’-GTGATGTCTGAACTGACCACGGCGTAGTCGTTTAACCCGTTG-3’ |
| *aguA*-3 | Forward primer for downstream of *aguA* | 5’-CAACGGGTTAAACGACTACGCCGTGGTCAGTTCAGACATCAC-3’ |
| *aguA*-4 | Reverse primer for downstream of *aguA* | 5’-CTTATGGTACCCGGGGATCCGGCATCTGCTGGGATCAATGGTTCC-3’ |
| *aguB*-1 | Forward primer for upstream of *aguB* | 5’-GTCGAC***ggatcc***CCGGGTACCTTGTTCAGGTGCGGATTGCG-3’ |
| *aguB*-2 | Reverse primer for upstream of *aguB* | 5’-CGCATGACAAAAGTTACCGTTGCCGTGATGTCTGAACTGACCACG-3’ |
| *aguB*-3 | Forward primer for downstream of *aguB* | 5’-CGTGGTCAGTTCAGACATCACGGCAACGGTAACTTTTGTCATGCG-3’ |
| *aguB*-4 | Reverse primer for downstream of *aguB* | 5’-CTTATGGTACCCGGGGATCCGTACTCCATCGAACTGCTGCAGC-3’ |
| *speC*-1 | Forward primer for upstream of *speC* | 5’-GTCGAC***ggatcc***CCGGGTACCGCGCGGTGTTGCGTCGATAAATAC-3’ |
| *speC*-2 | Reverse primer for upstream of *speC* | 5’-GTGCATACGGAACGAATCGATTAACGAGCCTGTTTCATGTAACCAAACCCAAACGG-3’ |
| *speC*-3 | Forward primer for downstream of *speC* | 5’-CCGTTTGGGTTTGGTTACATGAAACAGGCTCGTTAATCGATTCGTTCCGTATGCAC-3’ |
| *speC*-4 | Reverse primer for downstream of *speC* | 5’-CTTATGGTACCCGGGGATCCCCGCGCCGAAATTCCTCGGTAATATG-3’ |
| *speD*-1 | Forward primer for upstream of *speD* | 5’-GTCGAC***ggatcc***CCGGGTACGCTTTCGTCTGGTGATTGACGACGG-3’ |
| *speD*-2 | Reverse primer for upstream of *speD* | 5’-GCCCTCAGGTGAATGGTCAAACGGCAGCTTTTGCAATTTAATTCACCCCCTTATG-3’ |
| *speD*-3 | Forward primer for downstream of *speD* | 5’-CATAAGGGGGTGAATTAAATTGCAAAAGCTGCCGTTTGACCATTCACCTGAGGGC-3’ |
| *speD*-4 | Reverse primer for downstream of *speD* | 5’-CTTATGGTACCCGGGGATCCCATCGTTGTCTCAGGCTGTGACG-3’ |
| *speE*-1 | Forward primer for upstream of *speE* | 5’-GTCGAC***ggatcc***CCGGGTACGCCTCTTGCGGATATCACGCTGATC-3’ |
| *speE*-2 | Reverse primer for upstream of *speE* | 5’-CTTATGGATGCCGTGGTCAGCGCTGGGACATGTTAGGGCTTCC-3’ |
| *speE*-3 | Forward primer for downstream of *speE* | 5’-GGAAGCCCTAACATGTCCCAGCGCTGACCACGGCATCCATAAG-3’ |
| *speE*-4 | Reverse primer for downstream of *speE* | 5’-CTTATGGTACCCGGGGATCCCCAGCTGATGAATCAGATAGTTGAGCGC-3 |
| *potF*-1 | Forward primer for upstream of *potF* | 5’-GTCGAC***ggatcc***CCGGGTACGCAGGCTGAGGAGCAAATCTCCATG-3’ |
| *potF*-2 | Reverse primer for upstream of *potF* | 5’-CAAGGGTGGCGGTTATTTCCCGGGTGAACATCCGTTCCTTCCTCC-3’ |
| *potF*-3 | Forward primer for downstream of *potF* | 5’-GGAGGAAGGAACGGATGTTCACCCGGGAAATAACCGCCACCCTTG-3’ |
| *potF*-4 | Reverse primer for downstream of *potF* | 5’-CTTATGGTACCCGGGGATCCGCGACAGCTTGTCCTGTTTCAGGC-3’ |
| *plaP*-1 | Forward primer for upstream of *plaP* | 5’-GTCGAC***ggatcc***CCGGGTACGCAGGTGATAACCGAACTTGAACTGGATC-3’ |
| *plaP*-2 | Reverse primer for upstream of *plaP* | 5’-CATCGTCAGTCAGCGCATTACGCGGAATCAGTCATGACGCAGACCTCC-3’ |
| *plaP*-3 | Forward primer for downstream of *plaP* | 5’-GGAGGTCTGCGTCATGACTGATTCCGCGTAATGCGCTGACTGACGATG-3’ |
| *plaP*-4 | Reverse primer for downstream of *plaP* | 5’-CTTATGGTACCCGGGGATCCGCTCAGGTCCAGTTAGTCTCACCGTC-3’ |
| *pecS*-1 | Forward primer for upstream of *pecS* | 5’-GTCGAC***ggatcc***CCGGGTACCCGGCATCCATAACGAAGACAC-3’ |
| *pecS*-2 | Reverse primer for upstream of *pecS* | 5’-CAAGGATATCTAGTTAATGGCACGCCGTTAATGATCGGGTGACATCGC-3’ |
| *pecS*-3 | Forward primer for downstream of *pecS* | 5’-GCGATGTCACCCGATCATTAACGGCGTGCCATTAACTAGATATCCTTG-3’ |
| *pecS*-4 | Reverse primer for downstream of *pecS* | 5’-CTTATGGTACCCGGGGATCCGTGCGCCCAGTACAAACAAG-3’ |
| *cheB*-1 | Forward primer for upstream of *cheB* | 5’-GTCGAC***ggatcc***CCGGGTACCTTCAGTGATCGCCTGATCC-3’ |
| *cheB*-2 | Reverse primer for upstream of *cheB* | 5’-GGGCTGGCTAAGGAAAGATAATGAGCGTATATAGCAGCTCCGGGTGAG-3’ |
| *cheB*-3 | Forward primer for downstream of *cheB* | 5’-CTCACCCGGAGCTGCTATATACGCTCATTATCTTTCCTTAGCCAGCCC-3’ |
| *cheB*-4 | Reverse primer for downstream of *cheB* | 5’-CTTATGGTACCCGGGGATCCCCTTGCTTTGCTGGAATCGG-3’ |
| **Used for detection mutants** |  |  |
| *speA*-5 | Forward primer for detection of △*A* | 5’-CAGTTGCGTAATCAGGAAGGTGC-3’ |
| *speA*-6 | Reverse primer for detection of △*A* | 5’-CCGGTTGAAGATGTTCATCGGC-3’ |
| *aguA*-5 | Forward primer for detection of △*uA* | 5’-GCAGCCACCTTCAAAGAAATCGCC-3’ |
| *aguA*-6 | Reverse primer for detection of △*uA* | 5’-CCAGCCCGCTTATCAAACACTTCTC-3’ |
| *aguB*-5 | Forward primer for detection of △*uB* | 5’-GATTTTCAGCGACCGGCCTTG-3’ |
| *aguB*-6 | Reverse primer for detection of △*uB* | 5’-GTGGACGAACTGGCGCACTATATC-3’ |
| *speC*-5 | Forward primer for detection of △*C* | 5’-GTTACCCTCATTGGCAGCCG-3’ |
| *speC*-6 | Reverse primer for detection of △*C* | 5’-CAGCCTCAACTCCGGGCTGTATTC-3’ |
| *speD*-5 | Forward primer for detection of △*D* | 5’-GCGTTCTGCCGTCAGTATCTGC-3’ |
| *speD*-6 | Reverse primer for detection of △*D* | 5’-GACGAACCGATGCGTGTTTTCCG-3’ |
| *speE*-5 | Forward primer for detection of △*E* | 5’-GCCGGTAATTGCGCCATTGAAC-3’ |
| *speE*-6 | Reverse primer for detection of △*E* | 5’-CGTTGATGTCGCGGGTAAAACCG-3’ |
| *potF*-5 | Forward primer for detection of  △*F*, △*FP*, △*AF*, △*AFP* and △*ACFP* | 5’-GATCGTCTGCCAGACATTCAGC-3’ |
| *potF*-6 | Reverse primer for detection of  △*F*, △*FP*, △*AF*, △*AFP* and △*ACFP* | 5’-CACCAGCGTCAGCATCTCTTC-3’ |
| *plaP*-5 | Forward primer for detection of  △*P*, △*FP*, △*AP*, △*AFP* and △*ACFP* | 5’-GTTGACAGTAAAGGCGAGGTGAAGC-3’ |
| *plaP*-6 | Reverse primer for detection of  △*P*, △*FP*, △*AP*, △*AFP* and △*ACFP* | 5’-GCTGCTTTGTCGGCCATCATGTTAC-3’ |
| *pecS*-5 | Forward primer for detection of △*pecS* | 5’-GTTGCTGGAAGCCAATCGCATC-3’ |
| *pecS*-6 | Reverse primer for detection of △*pecS* | 5’-GCTGCGTCAGCAACAGGAAAG-3’ |
| *cheB*-5 | Forward primer for detection of △*cheB* | 5’-CAAACCACTGATCCCAGCGAAC-3’ |
| *cheB*-6 | Reverse primer for detection of △*cheB* | 5’-GAATCAGCCAGCTTATCTATCAGCGC-3’ |
| **Used for gene complementation** |  |  |
| C-*speA*-F | Forward primer of *speA* | 5’-GAATTCCCGG***ggatcc***CTTCCTGGTGATGTTTTCCCTGG-3’ |
| C-*speA*-R | Reverse primer of *speA* | 5’-CTTGGCTGCAGGTCGACTTATTCGTCTTCCAGATACGTATAACCG-3’ |
| C-*speC*-F | Forward primer of *speC* | 5’-GAATTCCCGG***ggatcc***CGTGCTGACGCTCCCTGTTAG-3’ |
| C-*speC*-R | Reverse primer of *speC* | 5’-CTTGGCTGCAGGTCGACTTAACGAGCAATCACATTCGCGC-3’ |
| C-*potF*-F | Forward primer of *potF* | 5’-GAATTCCCGG***ggatcc***TCGCGTTATGTATCAATAGCCGAC-3’ |
| C-*potF*-R | Reverse primer of *potF* | 5’-CTTGGCTGCAGGTCGACGCGGTTATTTCCCGCTTTTCAC-3’ |
| C-*plaP*-F | Forward primer of *plaP* | 5’-GAATTCCCGG***ggatcc***AGCCGTTTGATTCGTGCC-3’ |
| C-*plaP*-R | Reverse primer of *plaP* | 5’-CTTGGCTGCAGGTCGACGCGCATTACGCCAGATCTTC-3’ |
| **Used for plasmid detection** |  |  |
| pKNG101-F | Forward primer of pKNG101 | 5’-CATATCACAACGTGCGTGG-3’ |
| pKNG101-R | Reverse primer of pKNG101 | 5’-CTGCCGATGTCCAGTAAGATTC-3’ |
| PLAFR3-F | Forward primer of PLAFR3 | 5’-GACAGGTTTCCCGACTGGAAAGC-3’ |
| PLAFR3-R | Reverse primer of PLAFR3 | 5’-CGAAAGGGGGATGTGCTGCAAG-3’ |
| **Used for qRT-PCR** |  |  |
| Q-*infB*-F | Forward primer of *infB* rDNA | 5’-CTCGTTGCTGGACTACAT-3’ |
| Q-*infB*-R | Reverse primer of *infB* rDNA | 5’-GGTAATCATGCCGTTGTC-3’ |
| Q-*Pecs*-F | Forward primer of *Pecs* rDNA | 5’-CACGCTACCTGGAAGTAT-3’ |
| Q-*Pecs*-R | Reverse primer of *Pecs* rDNA | 5’-CGCTCAGTTTGTACTTGC-3’ |
| Q-*bglA*-F | Forward primer of *bglA* rDNA | 5’-GGGCTACCACATCCATAT-3’ |
| Q-*bglA-*R | Reverse primer of *bglA* rDNA | 5’-GGCGTCAATATCGTTCTG-3’ |
| Q-*bgxA*-F | Forward primer of *bgxA* rDNA | 5’-TTCCAGACCGGGTTATTC-3’ |
| Q-*bgxA-*R | Reverse primer of *bgxA* rDNA | 5’-GGCAGGAGATTGTTGTTC-3’ |
| Q-*nagZ*-F | Forward primer of *nagZ* rDNA | 5’-GGCATCATCTTCTCGGAT-3’ |
| Q-*nagZ-*R | Reverse primer of *nagZ* rDNA | 5’-CCTGACGCTGGTTACATA-3’ |
| Q-*celZ*-F | Forward primer of *celZ* rDNA | 5’-CGCTTCAGGTATCATGGA-3’ |
| Q-*celZ-*R | Reverse primer of *celZ* rDNA | 5’-CGTGCCGACAATAATCAG-3’ |
| Q-*celY*-F | Forward primer of *celY* rDNA | 5’-CGACAACTGATTGACGAC-3’ |
| Q-*celY-*R | Reverse primer of *celY* rDNA | 5’-AGGTACAACGGAATACGG-3’ |
| Q-*pelB*-F | Forward primer of *pelB* rDNA | 5’-CCGACTTCTCTACCTACAG-3’ |
| Q-*pelB-*R | Reverse primer of *pelB* rDNA | 5’-GCGTAGTTAGCCAGTTTG-3’ |
| Q-*pelC*-F | Forward primer of *pelC* rDNA | 5’-CTCAGGATGGCGATATGT-3’ |
| Q-*pelC-*R | Reverse primer of *pelC* rDNA | 5’-GATGTCAAAGGCGGATTC-3’ |
| Q-*pelD*-F | Forward primer of *pelD* rDNA | 5’-TCCAACCTGAAGAGCATC-3’ |
| Q-*pelD-*R | Reverse primer of *pelD* rDNA | 5’-CATGGTCTGAGCCGAATA-3’ |
| Q-*pelE*-F | Forward primer of *pelE* rDNA | 5’-GTGACCTTCCACAACAAC-3’ |
| Q-*pelE-*R | Reverse primer of *pelE* rDNA | 5’-CTGTACTGATAGCGGTAGG-3’ |
| Q-*pelI*-F | Forward primer of *pelI* rDNA | 5’-GCGTCAGGAAGTTTATCG-3’ |
| Q-*pelI-*R | Reverse primer of *pelI* rDNA | 5’-CCAGTACCAGTAGTTGAGG-3’ |
| Q-*pelZ*-F | Forward primer of *pelZ* rDNA | 5’-GCCTATAACGTGGTGGTA-3’ |
| Q-*pelZ-*R | Reverse primer of *pelZ* rDNA | 5’-GTGAATGCCTTTGGAGTG-3’ |
| Q-*pelX*-F | Forward primer of *pelX* rDNA | 5’-GCAGCTACAACCAGATTG-3’ |
| Q-*pelX-*R | Reverse primer of *pelX* rDNA | 5’-TCCGAGTTCAGAATCAGG-3’ |
| Q-*prtA*-F | Forward primer of *prtA* rDNA | 5’-GTGACCTATGCCGAAGAT-3’ |
| Q-*prtA-*R | Reverse primer of *prtA* rDNA | 5’-AAGCCATAGACGGTATCG-3’ |
| Q-*prtB*-F | Forward primer of *prtB* rDNA | 5’-CGTCAGTTCAGCATCATG-3’ |
| Q-*prtB-*R | Reverse primer of *prtB* rDNA | 5’-CGGTGTTGGAGTTAAAGC-3’ |
| Q-*prtC*-F | Forward primer of *prtC* rDNA | 5’-TACAGGATCAGTTCACCG-3’ |
| Q-*prtC-*R | Reverse primer of *prtC* rDNA | 5’-CGATTCGTACCAGGAAGT-3’ |
| Q-*prtG*-F | Forward primer of *prtG* rDNA | 5’-CGCCAGTTCAGCATTATG-3’ |
| Q-*prtG-*R | Reverse primer of *prtG* rDNA | 5’-GACCGCTATTGGAGTGAA-3’ |
| Q-*cheD*-F | Forward primer of *cheD* rDNA | 5’-AGGACAAGTTCGAGAAGG-3’ |
| Q-*cheD-*R | Reverse primer of *cheD* rDNA | 5’-ACAACCAGTATCGCTACC-3’ |
| Q-*cheR*-F | Forward primer of *cheR* rDNA | 5’-AAGCGTGAGATGGTCTAC-3’ |
| Q-*cheR-*R | Reverse primer of *cheR* rDNA | 5’-CCGTCAGATTGGTCGTTA-3’ |
| Q-*cheB*-F | Forward primer of *cheB* rDNA | 5’-ATCGTCATCGTCCTTCTG-3’ |
| Q-*cheB-*R | Reverse primer of *cheB* rDNA | 5’-GGCATACCGAATACCACA-3’ |
| Q-*cheA*-F | Forward primer of *cheA* rDNA | 5’-CATTCTTCTGCCGTTGAC-3’ |
| Q-*cheA-*R | Reverse primer of *cheA* rDNA | 5’-CCAGCCAGAGGATACAAA-3’ |
| Q-*cheW*-F | Forward primer of *cheW* rDNA | 5’-AATTAGCAGGCGAGACAG-3’ |
| Q-*cheW-*R | Reverse primer of *cheW* rDNA | 5’-GTGTTGGCAATACGAGTG-3’ |
| Q-*cheZ*-F | Forward primer of *cheZ* rDNA | 5’-CAACTGCTGATGGTCTTG-3’ |
| Q-*cheZ-*R | Reverse primer of *cheZ* rDNA | 5’-GGCTATCCAACAGGTCAT-3’ |
| Q-*cheY*-F | Forward primer of *cheY* rDNA | 5’-GATGCGTCGTATTGTTCG-3’ |
| Q-*cheY-*R | Reverse primer of *cheY* rDNA | 5’-GCTCAAGTCCGTCCATAT-3’ |
| Q-*fliM*-F | Forward primer of *fliM* rDNA | 5’-CACCAACATCACCACATC-3’ |
| Q-*fliM-*R | Reverse primer of *fliM* rDNA | 5’-GGATTAGCCAGCAATTCG-3’ |
| Q-*fliN*-F | Forward primer of *fliN* rDNA | 5’-GAAACCGTCCGAAGAAAC-3’ |
| Q-*fliN-*R | Reverse primer of *fliN* rDNA | 5’-GTGTCCTGCACATCAAAG-3’ |
| Q-*motA*-F | Forward primer of *motA* rDNA | 5’-GTATCAGCCTTCGGAACT-3’ |
| Q-*motA-*R | Reverse primer of *motA* rDNA | 5’-GAACAATACCGCCATCAG-3’ |
| Q-*motB*-F | Forward primer of *motB* rDNA | 5’-GATTGCCATTTCCTCACC-3’ |
| Q-*motB-*R | Reverse primer of *motB* rDNA | 5’-CTTCACATCGCCTTCTTG-3’ |
| Q-*rpoD*-F | Forward primer of *rpoD* rDNA | 5’-ACAAGTTCTCCACCTACG-3’ |
| Q-*rpoD-*R | Reverse primer of *rpoD* rDNA | 5’-GCGGGAAATACGATTGAG-3’ |
| Q-*flhC*-F | Forward primer of *flhC* rDNA | 5’-GGCGATGGAACTCATTTC-3’ |
| Q-*flhC-*R | Reverse primer of *flhC* rDNA | 5’-CGGCAACATTCCTTTAGG-3’ |
| Q-*flhD*-F | Forward primer of *flhD* rDNA | 5’-CCTCTGAATTGCTCAAGC-3’ |
| Q-*flhD-*R | Reverse primer of *flhD* rDNA | 5’-ATACCCAACCGGAACATC-3’ |
| Q-*fliA*-F | Forward primer of *fliA* rDNA | 5’-ACTGTATACCGCTGAAGG-3’ |
| Q-*fliA-*R | Reverse primer of *fliA* rDNA | 5’-CGGAAGACGAACCTGTAA-3’ |
| Q-*flgM*-F | Forward primer of *flgM* rDNA | 5’-GGTTAAACTGAGCGATGC-3’ |
| Q-*flgM-*R | Reverse primer of *flgM* rDNA | 5’-CCTGCGATTAAGTCCTGA-3’ |
| Q-*flgN*-F | Forward primer of *flgN* rDNA | 5’-GTTGTGCTGTCTGATGAG-3’ |
| Q-*flgN-*R | Reverse primer of *flgN* rDNA | 5’-CTCGCAACACTGATTCTC-3’ |
| Q-*flhA*-F | Forward primer of *flhA* rDNA | 5’-TGATTGGTGAGGATGAGG-3’ |
| Q-*flhA-*R | Reverse primer of *flhA* rDNA | 5’-CGCCGATGATGTTAATGG-3’ |
| Q-*flhB*-F | Forward primer of *flhB* rDNA | 5’-GCGGATGTGATTGTTACC-3’ |
| Q-*flhB-*R | Reverse primer of *flhB* rDNA | 5’-TTCCAGAATCGGGATACG-3’ |
| Q-*fliZ*-F | Forward primer of *fliZ* rDNA | 5’-CAAGCAACCAGGAAGAAG-3’ |
| Q-*fliZ-*R | Reverse primer of *fliZ* rDNA | 5’-CGAAACACGATCCAGTTC-3’ |
| Q-*fliC*-F | Forward primer of *fliC* rDNA | 5’-CGTAGCCGTATTCAGGAT-3’ |
| Q-*fliC-*R | Reverse primer of *fliC* rDNA | 5’-TCTGCGGTACTTGGTTAG-3’ |
| Q-*fliD*-F | Forward primer of *fliD* rDNA | 5’-GGTTAGCAGCAACTCAAG-3’ |
| Q-*fliD-*R | Reverse primer of *fliD* rDNA | 5’-TAAGGCGTCAATCTCTGG-3’ |
| Q-*fliS*-F | Forward primer of *fliS* rDNA | 5’-GTCCGCATCAACTGATTG-3’ |
| Q-*fliS-*R | Reverse primer of *fliS* rDNA | 5’-CCATATCCAGTCCGAGTT-3’ |
| Q-*fliT*-F | Forward primer of *fliT* rDNA | 5’-TGGATACGGAATCACAGG-3’ |
| Q-*fliT-*R | Reverse primer of *fliT* rDNA | 5’-TGGAGAACTCGGCATAAG-3’ |
| Q-*fliQ*-F | Forward primer of *fliQ* rDNA | 5’-GACTCCTGAATCCGTCAT-3’ |
| Q-*fliQ-*R | Reverse primer of *fliQ* rDNA | 5’-TACCGTCAGCACTTTAGG-3’ |
| Q-*ycgR*-F | Forward primer of *ycgR* rDNA | 5’-TCTGGGACACTGAATGAC-3’ |
| Q-*ycgR-*R | Reverse primer of *ycgR* rDNA | 5’-CATTACTGTGGCGTCTTG-3’ |

Note: BamHI cleavage site is shown in lower case, italic and bold font.

**Table S4.** Similarity of *D. fangzhongdai* ZXC1 putative polyamine transporter substrate-binding protein PotD with its homologs in other bacterial species.

| Strains  (Sequence accession number in NCBI) | Amino acid identity to the PotD homologs from strain ZXC1 (%) | | |
| --- | --- | --- | --- |
|  | PotD1 | PotD2 | PotD3 |
| *Dickeya dadantii* 3937  (CP002038.1) | 98.29 | 90.18 | 96.50 |
| *Dickeya oryzae* EC1  (CP006929.1) | 97.14 | 88.01 | 95.34 |
| *Escherichia coli* str. K-12 MG 1655  (NC_000913.3) | - | 41.18 | 28.00 |
| *Yersinia pestis* CO92  (CP009973.1) | - | 39.30 | - |
| *Yersinia pestis* KIM10+  (AE009952.1) | 23.65 | - | - |
| *Pseudomonas aeruginosa* PAO1 (NC_002516.2) | 23.76 | 25.52 | 22.01 |

-Note: “-” indicates the gene was not fund in the corresponding genome.

**Table S5.** Analysis of the genes encoding PCWD enzymes, motility and corresponding regulators in *D. fangzhongdai* ZXC1.

| Gene | Sequence accession number in NCBI | Products | Amino acid identity with homolog  in *D. dadantii* 3937 (%) | |
| --- | --- | --- | --- | --- |
| *pecS* | ADN00620.1 | Negative regulator of pectinase, cellulase, blue pigment production and virulence genes | | 99.40 |
| *bglA* | ADM96608.1 | 6-phospho-β-glucosidase | | 94.98 |
| *bgxA* | ADM98002.1 | β-glucosidase (Gentiobiase)  (Cellobiase)/β-xylosidase | | 95.44 |
| *nagZ* | ADM98979.1 | β-hexosaminidase | | 96.77 |
| *celZ* | ADM99099.1 | Endoglucanase | | 93.67 |
| *celY* | ADM96331.1 | Minor endoglucanase | | 94.60 |
| *pelB* | ADN00345.1 | Pectin lyase | | 93.87 |
| *pelC* | ADN00346.1 | Pectate lyase | | 95.47 |
| *pelD* | ADM99552.1 | Pectate lyase | | 93.62 |
| *pelE* | ADM99551.1 | Pectate lyase | | 96.29 |
| *pelI* | ADM99410.1 | Pectate lyase | | 97.38 |
| *pelZ* | ADN00347.1 | Pectate lyase | | 94.05 |
| *pelX* | ADN00761.1 | Exopolygalacturonate lyase | | 98.37 |
| *prtA* | ADM98496.1 | Serralysin family metalloprotease | | 97.99 |
| *prtB* | ADM98498.1 | Serralysin family metalloprotease | | 97.51 |
| *prtC* | ADM98497.1 | Serralysin family metalloprotease | | 98.49 |
| *prtG* | ADM98503.1 | Serralysin family metalloprotease | | 95.39 |
| *cheD* | ADM99083.1 | methyl-accepting chemotaxis protein | | 97.18 |
| *cheR* | ADM99082.1 | Chemotaxis protein methyltransferase | | 100.00 |
| *cheB* | ADM99081.1 | Chemotaxis response regulator protein-glutamate methylesterase | | 100.00 |
| *cheA* | ADM99085.1 | Chemotaxis protein | | 94.13 |
| *cheW* | ADM99084.1 | Positive regulator of CheA protein activity | | 100.00 |
| *cheZ* | ADM99079.1 | Chemotaxis response-phosphatase | | 100.00 |
| *cheY* | ADM99080.1 | Chemotaxis regulator-transmits chemoreceptor signals to  Flagellar motor components | | 100.00 |
| *fliM* | ADM99055.1 | Flagellar motor switch protein | | 99.11 |
| *fliN* | ADM99056.1 | Flagellar motor switch protein | | 99.28 |
| *motA* | ADM99087.1 | Flagellar motor rotation protein | | 98.98 |
| *motB* | ADM99086.1 | Flagellar motor rotation protein | | 98.67 |
| *rpoD* | ADM96819.1 | RNA polymerase, sigma 70 factor | | 98.36 |
| *flhC* | ADM99088.1 | Flagellar transcriptional activator | | 98.44 |
| *flhD* | ADM99089.1 | Flagellar transcriptional activator | | 99.14 |
| *fliA* | ADM99035.1 | RNA polymerase, sigma 28 factor | | 99.58 |
| *flgM* | ADM99073.1 | FliA specific anti-sigma factor | | 98.99 |
| *flgN* | ADM99074.1 | Flagellar biosynthesis protein | | 96.97 |
| *flhA* | ADM99076.1 | flagellar export pore protein | | 98.56 |
| *flhB* | ADM99077.1 | Flagellar biosynthesis protein | | 97.39 |
| *fliZ* | ADM.99034.1 | regulator of FliA activity | | 99.41 |
| *fliC* | ADM99043.1 | flagellar filament structural protein | | 95.36 |
| *fliD* | ADM99044.1 | Flagellar hook-associated protein | | 83.30 |
| *fliS* | ADM99045.1 | Flagellar biosynthesis protein | | 98.53 |
| *fliT* | ADM99046.1 | Flagellar biosynthesis protein | | 98.33 |
| *fliQ* | ADM99059.1 | Flagellar biosynthesis protein | | 100.00 |
| *ycgR* | ADM98288.1 | c-di-GMP-binding flagellar brake protein | | 94.49 |


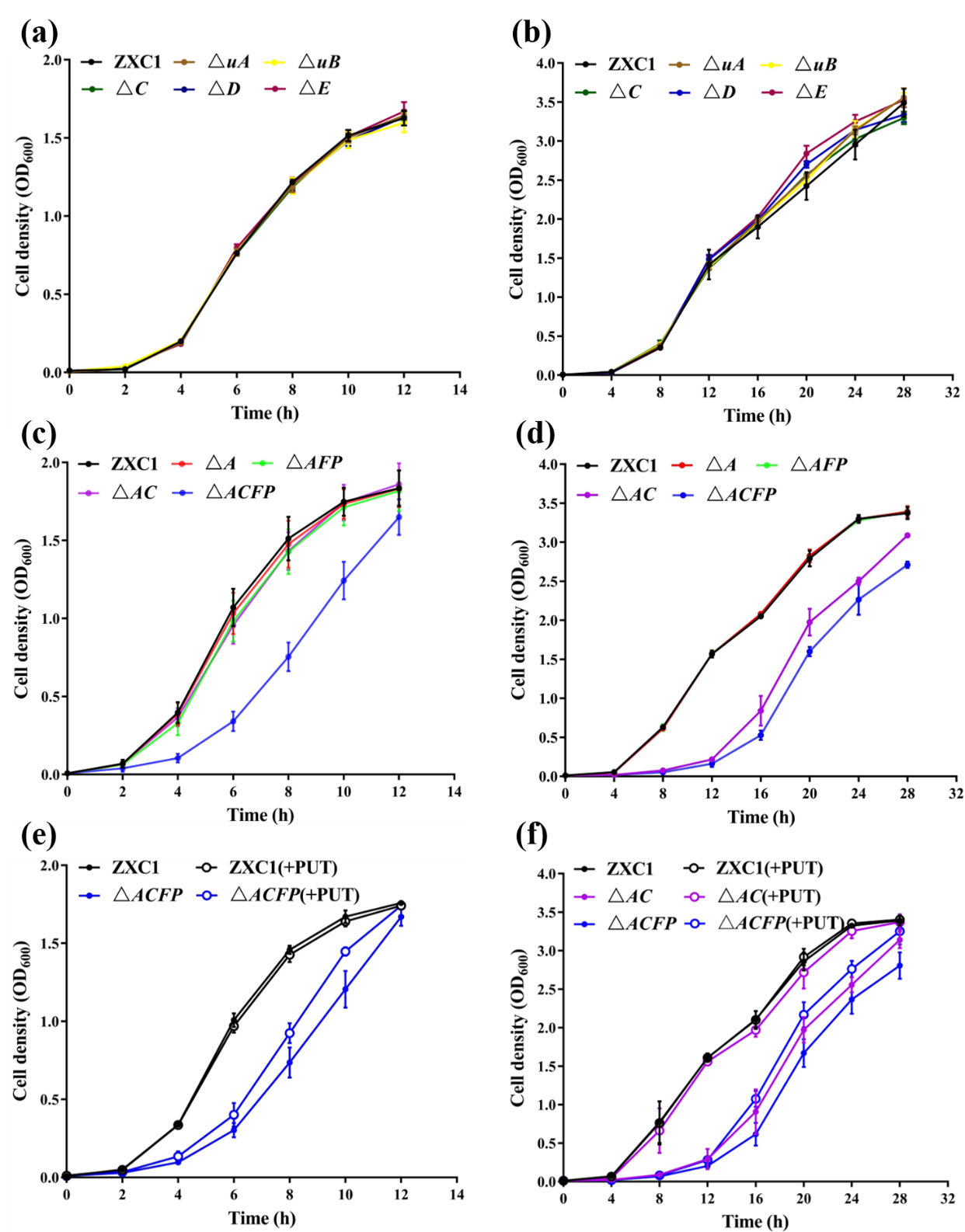


**Fig. S1** Growth curves of wild-type *D. fangzhongdai* ZXC1 and its derivatives in LB or MM medium (with or without 0.1 mM putrescine). (a) Growth curves of *D. fangzhongdai* ZXC1 and single mutant△*uA*, △*uB*, △*C*, △*D*, and △*E* in LB medium; (b) Growth curves of *D. fangzhongdai* ZXC1 and single mutant△*uA*, △*uB*, △*C*, △*D*, and △*E* in MM medium; (c) Growth curves of *D. fangzhongdai* ZXC1, single mutant△*A*, double mutant △*AC*, triple mutant△*AFP*, and quadruple mutant△*ACFP* in LB medium; (d) Growth curves of *D. fangzhongdai* ZXC1, single mutant△*A*, double mutant △*AC*, triple mutant△*AFP*, and quadruple mutant△*ACFP* in MM medium; (e) Growth curves of *D. fangzhongdai* ZXC and quadruple mutant△*ACFP* in LB medium with or without 0.1 mM putrescine; (f) Growth curves of *D. fangzhongdai* ZXC double mutant △*AC* and quadruple mutant△*ACFP* in MM medium with or without 0.1 mM putrescine. Symbol: △*uA* is the in-frame deletion mutant of *aguA*, △*uB* is the in-frame deletion mutant of *aguB*, △*C* is the in-frame deletion mutant of *speC*, △*D* is the in-frame deletion mutant of *speD*, △*E* is the in-frame deletion mutant of *speE*, △*A* is the in-frame deletion mutant of *speA*, △*AC* is the double deletion mutant of *speA* and *speC*, △*AFP* is the tripple deletion mutant of *speA*, *potF* and *plaP*, △*ACFP* is quadruple deletion mutant of *speA*, *speC*, *potF* and *plaP*. The data shown are the mean ± SE (*n* = 3 independent experiments).


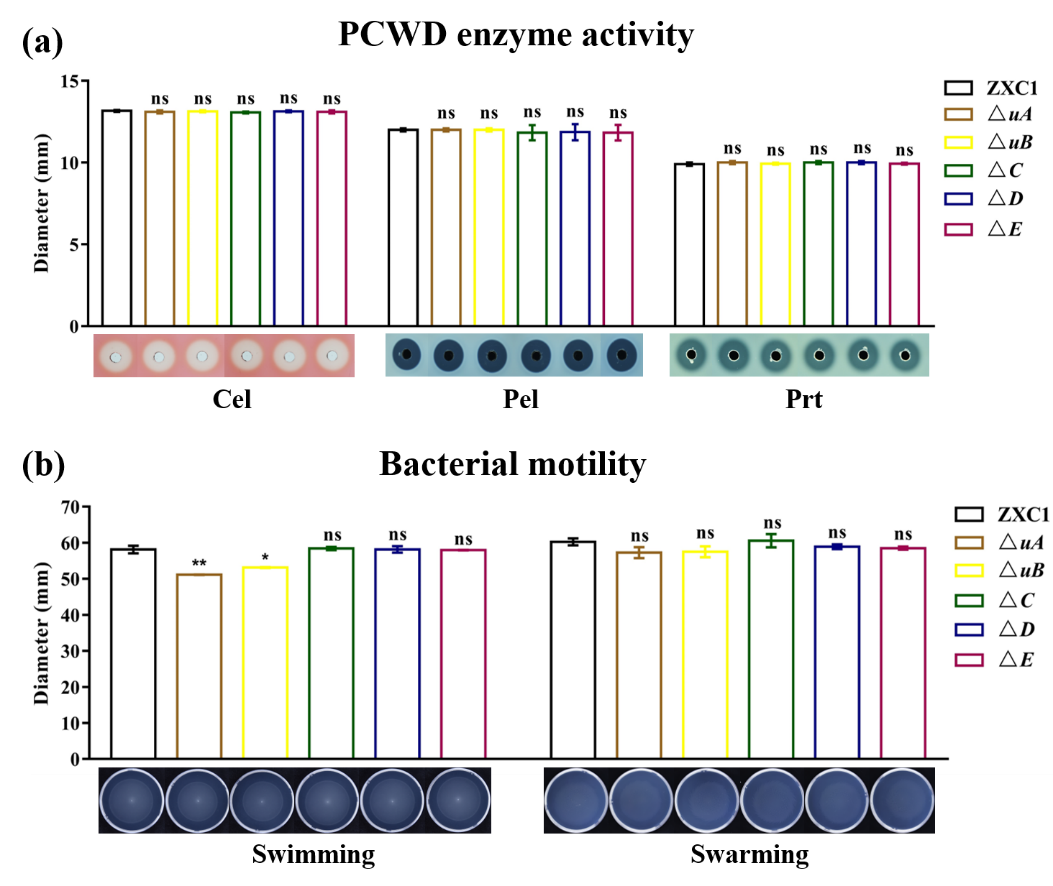


**Fig. S2** Analysis of PCWD enzyme activity and bacterial motility in wild-type *D. fangzhongdai* ZXC1 and polyamines synthesis genes mutants. (a) Analysis of PCWD enzyme activity in wild-type *D. fangzhongdai* ZXC1 and polyamines synthesis genes mutants; (b) Analysis of bacterial motility in wild-type *D. fangzhongdai* ZXC1 and polyamines synthesis genes mutants. The data shown are the mean ± SE (*n* = 3 independent experiments), ** *P*＜0.01, * *P*＜0.05, ns, not significant (by one-way analysis of variance [ANOVA] with multiple comparisons).


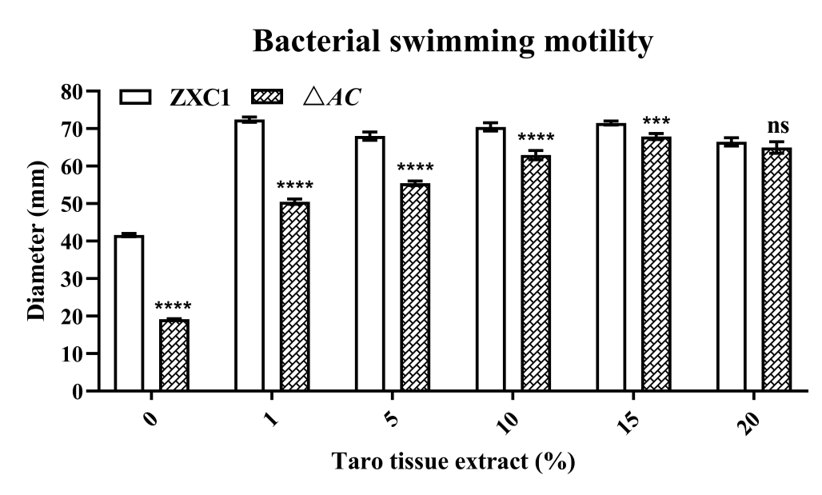


**Fig. S3** Effect of taro tissue extract on the swimming motility of *D. fangzhongdai* ZXC1 and double mutant△*AC*. The data shown are the mean ± SE (*n* = 3 independent experiments). The bacterial swimming motility with or without taro extract was recorded 10 h post inoculation. The experiments were repeated three times with triplicates. Statistical significance: ****, *P*＜0.0001; ***, *P*＜0.001; ns, not significant (by two-way analysis of variance [ANOVA] with multiple comparisons).


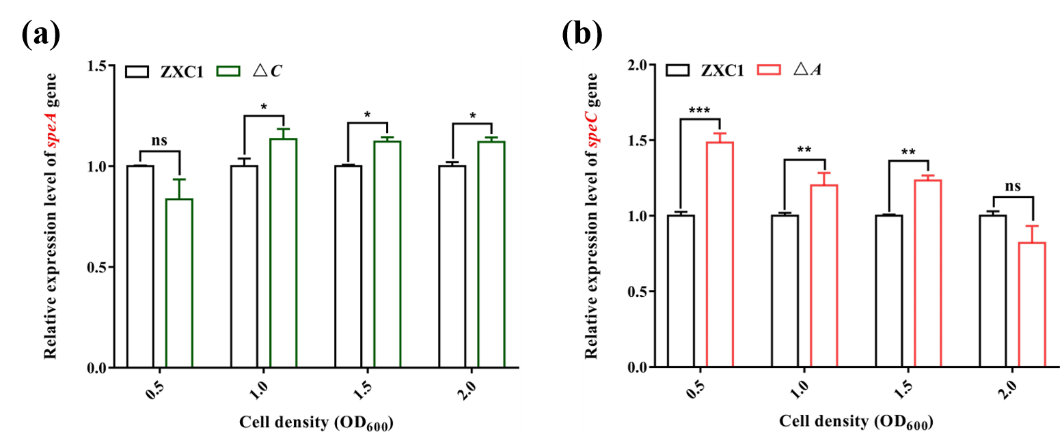


**Fig. S4** qRT-PCR analysis of *speA* or *speC* transcript level in the background of mutant △*C* (a) or △*A* (b), respectively. The experiments were repeated three times with triplicates. Statistical significance: ***, *P*＜0.001; **, *P*＜0.01; *, *P*＜0.05; ns, not significant (by Student’s *t* test).


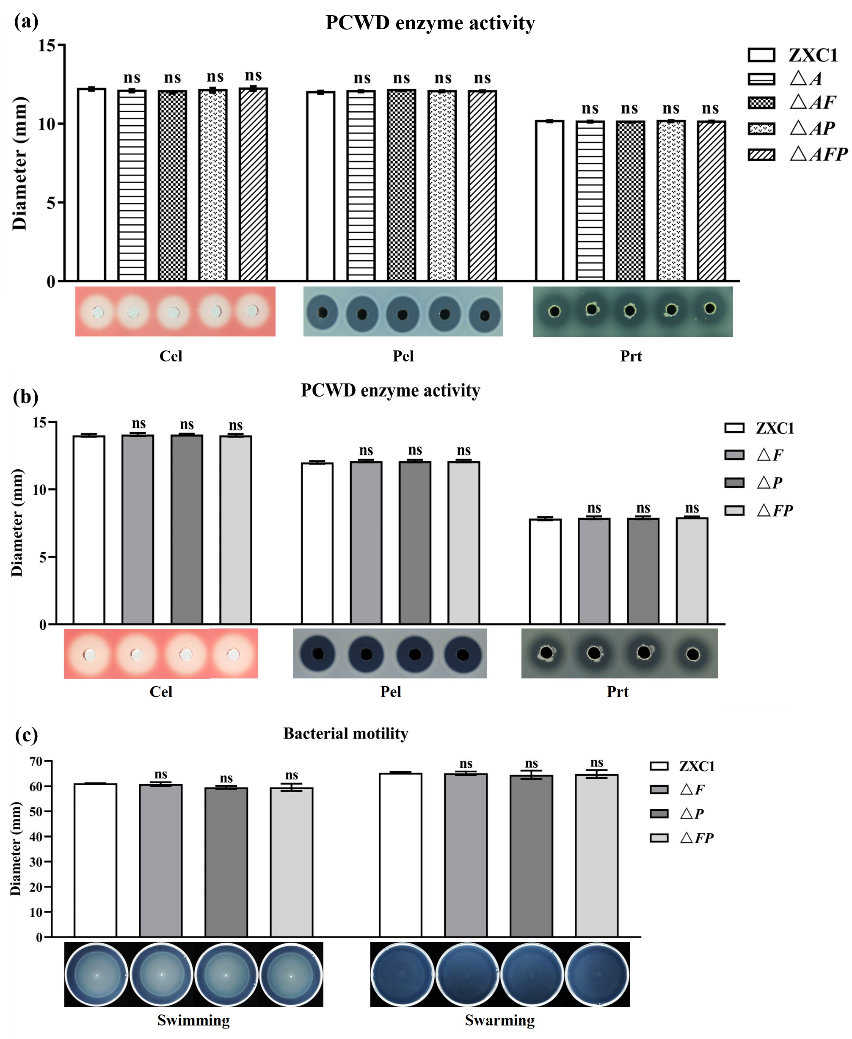


**Fig. S5** Analysis of PCWD enzyme activity and bacterial motility in wild-type *D. fangzhongdai* ZXC1 and mutant △*A*, △*AF*, △*AP*, △*AFP*, △*F*, △*P* and △*FP*. (a) Analysis of PCWD enzyme activity in wild-type *D. fangzhongdai* ZXC1 and mutant △*A*, △*AF*, △*AP* and △*AFP*; (b) Analysis of PCWD enzyme activity in wild-type *D. fangzhongdai* ZXC1 and mutant △*F*, △*P* and △*FP*; (c) Analysis of bacterial motility in wild-type *D. fangzhongdai* ZXC1 and mutant △*F*, △*P* and △*FP*. Symbol: △*A* is the in-frame deletion mutant of *speA*, △*AF* is the double deletion mutant of *speA* and *potF*, △*AP* is the double deletion mutant of *speA* and *plaP*, △*AFP* is the triple deletion mutants of *speA*, *potF* and *plaP*, △*F* is in-frame deletion mutant of *potF*, △*P* is the in-frame deletion mutant of *plaP*, △*FP* is double deletion mutant of *potF* and *plaP*. The data shown are the mean ± SE (*n* = 3 independent experiments), ns, not significant (by Student’s *t* test).


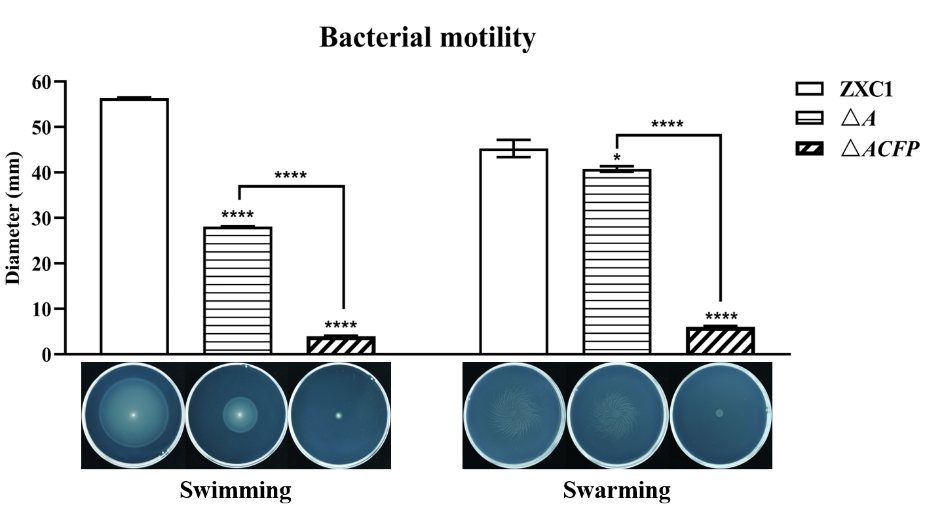


**Fig. S6** Bacterial motility assay of wild-type *D. fangzhongdai* ZXC1and mutant △*A* and quadruple mutant △*ACFP*. The data shown are the mean ± SE (*n* = 3 independent experiments). Statistical significance: ****, *P*＜0.0001 (by Student’s *t* test).


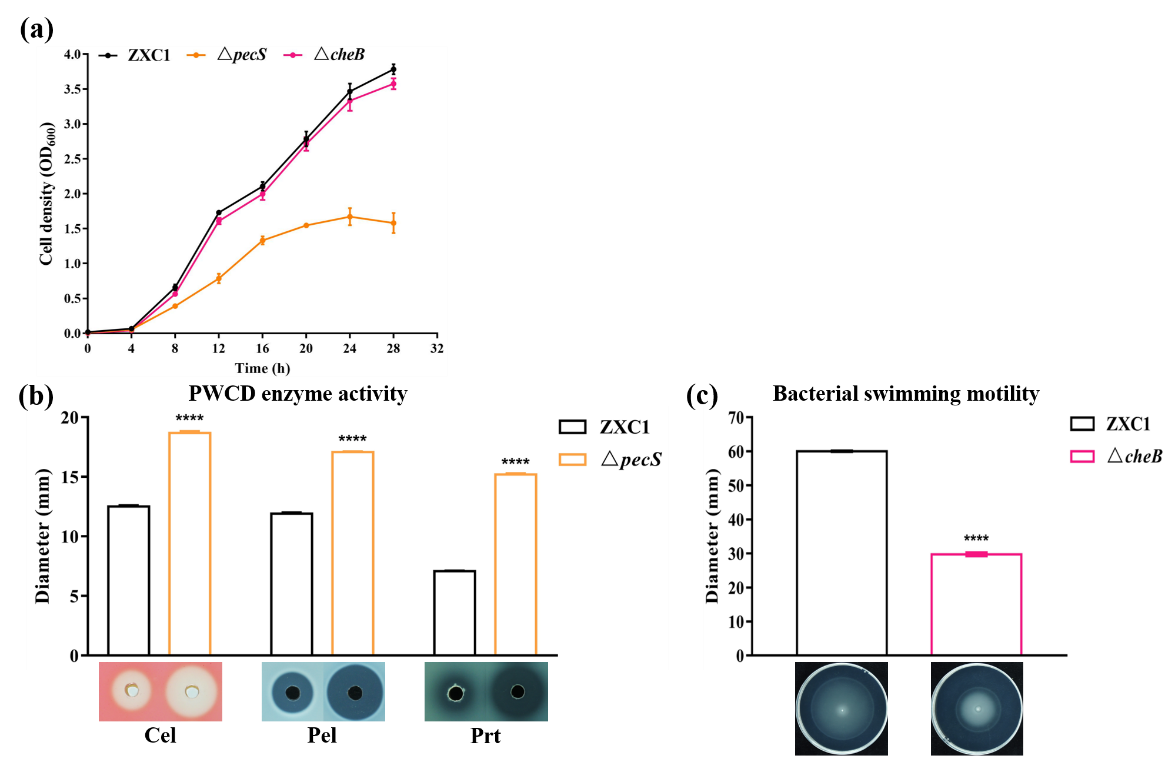


**Fig. S7** Impact of *pecS* and *cheB* mutation on *D. fangzhongdai* ZXC1 growth, PCWD enzyme production and motility. (a) Growth curves of strain ZXC1 and mutant (△*pecS* and △*cheB*) in MM medium; (b) PCWD enzyme activity of strain ZXC1 and mutant △*pecS*; (c) Bacterial motility of strain ZXC1 and mutant △*cheB*. △*pecS* is the in-frame deletion mutants of gene *pecS*, △*cheB* is double deletion mutants of gene *cheB.* The experiments were repeated at least three times with triplicates (*n* = 3 independent experiments). Statistical significance: ****, *P*＜0.0001 (by Student’s *t* test).

**
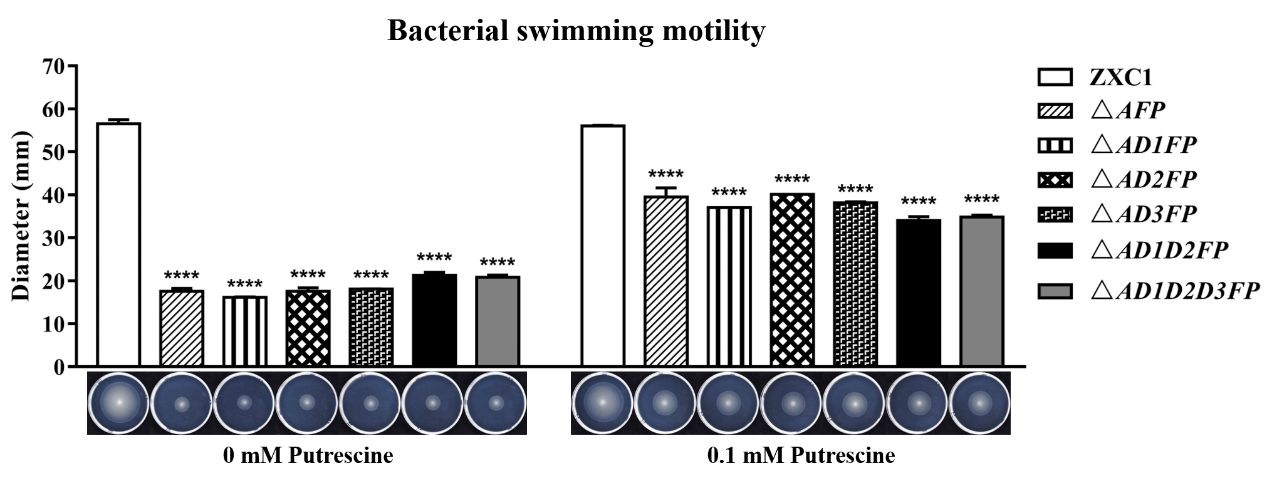
**

**Fig. S8** Analysis bacterial swimming motility in wild-type *D. fangzhongdai* ZXC1 and putative polyamine transporter substrate-binding protein PotD mutants with or without 0.1 mM putrescine. Symbol: △*AFP* is the tripple deletion mutant of *speA*, *potF* and *plaP*, △*AD1FP* is the quadruple deletion mutant of *speA*, *potD1*, *potF* and *plaP*, △*AD2FP* is quadruple deletion mutant of *speA*, *potD2*, *potF* and *plaP*, △*AD3FP* is the quadruple deletion mutant of *speA*, *potD3*, *potF* and *plaP*, △*AD1D2FP* is the quadruple deletion mutant of *speA*, *potD1*, *potD2*, *potF* and *plaP*, △*AD1D2D3FP* is the sextuple deletion mutant of *speA*, *potD1*, *potD2*, *potD3*, *potF* and *plaP*. The data shown are the mean ± SE (*n* = 3 independent experiments). Statistical significance: **** *P*＜0.0001 (Student’s *t* test).
